# Spores of *Clostridioides difficile* are toxin delivery vehicles

**DOI:** 10.1038/s42003-024-06521-x

**Published:** 2024-07-10

**Authors:** Carolina P. Cassona, Sara Ramalhete, Khira Amara, Thomas Candela, Imad Kansau, Cécile Denève-Larrazet, Claire Janoir-Jouveshomme, Luís Jaime Mota, Bruno Dupuy, Mónica Serrano, Adriano O. Henriques

**Affiliations:** 1 Instituto de Tecnologia Química e Biológica, NOVA University Lisbon, Oeiras, Portugal; 2 Micalis Institute, Université Paris-Saclay, INRAE, AgroParisTech, Jouy-en-Josas, France; 3 Associate Laboratory i4HB, Institute for Health and Bioeconomy, NOVA School of Science and Technology, NOVA University Lisbon, Caparica, Portugal; 4 UCIBIO, Applied Molecular Biosciences Unit, Department of Life Sciences, NOVA School of Science and Technology, NOVA University Lisbon, Caparica, Portugal; 5 Institut Pasteur, Université Paris-Cité, UMR-CNRS 6047, Laboratoire Pathogenèse des Bactéries Anaérobies, F-75015 Paris, France

**Keywords:** Pathogens, Microbiology

## Abstract

*Clostridioides difficile* causes a wide range of intestinal diseases through the action of two main cytotoxins, TcdA and TcdB. Ingested spores germinate in the intestine establishing a population of cells that produce toxins and spores. The pathogenicity locus, PaLoc, comprises several genes, including those coding for TcdA/B, for the holin-like TcdE protein, and for TcdR, an auto-regulatory RNA polymerase sigma factor essential for *tcdA*/*B* and *tcdE* expression. Here we show that *tcdR*, *tcdA*, *tcdB* and *tcdE* are expressed in a fraction of the sporulating cells, in either the whole sporangium or in the forespore. The whole sporangium pattern is due to protracted expression initiated in vegetative cells by σ^D^, which primes the TcdR auto-regulatory loop. In contrast, the forespore-specific regulatory proteins σ^G^ and SpoVT control TcdR production and *tcdA/tcdB* and *tcdE* expression in this cell. We detected TcdA at the spore surface, and we show that wild type and Δ*tcdA* or Δ*tcdB* spores but not Δ*tcdR* or Δ*tcdA/*Δ*tcdB* spores are cytopathic against HT29 and Vero cells, indicating that spores may serve as toxin-delivery vehicles. Since the addition of TcdA and TcdB enhance binding of spores to epithelial cells, this effect may occur independently of toxin production by vegetative cells.

## Abstract (web summary)

This study shows that the genes coding for the two main toxins produced by *Clostridioides difficile* are expressed during sporulation and that the toxins associate with the mature spores, which have a cytopathic effect on human intestinal cell lines.

## Introduction

*Clostridioides difficile* is a major nosocomial pathogen and the leading cause of intestinal diseases that range from mild diarrhea to life-threatening illnesses, linked to the use of antibiotics^1,2^. In the last two decades, the emergence and spreading of epidemic clones of ribotype 027 was responsible for worldwide outbreaks associated with more severe disease symptoms, recurrence rates, morbidity and mortality^1,2^. The epidemiology of *C. difficile* is however changing, with new ribotypes disseminating both in healthcare units and at the community level, and with increased incidence among groups not previously considered at risk^3^. Moreover, the prevalence of some ribotypes in animals used for human consumption raises serious concerns of widespread dissemination through the food chain^4,5^.

Infection is initiated by the ingestion of spores when gut dysbiosis, most frequently due to continued antibiotic treatment, allows spores to germinate in the intestine^6,7^. For many strains, the resulting cells produce two cytotoxins, TcdA and TcdB, which are the main factors responsible for the disease symptoms, and spores^1,2,8^. Spores are highly resistant dormant cells, hard to eradicate, that allows dissemination of this strict anaerobic pathogen^6,7,9,10^. Spores also allow *C. difficile* to persist in the environment and in the host and are linked to disease recurrence^6,7,11,12^. A recent study shows that spores enter intestinal epithelial cells and may persist in this intracellular niche contributing to disease recurrence^13^.

**Fig. 1:**
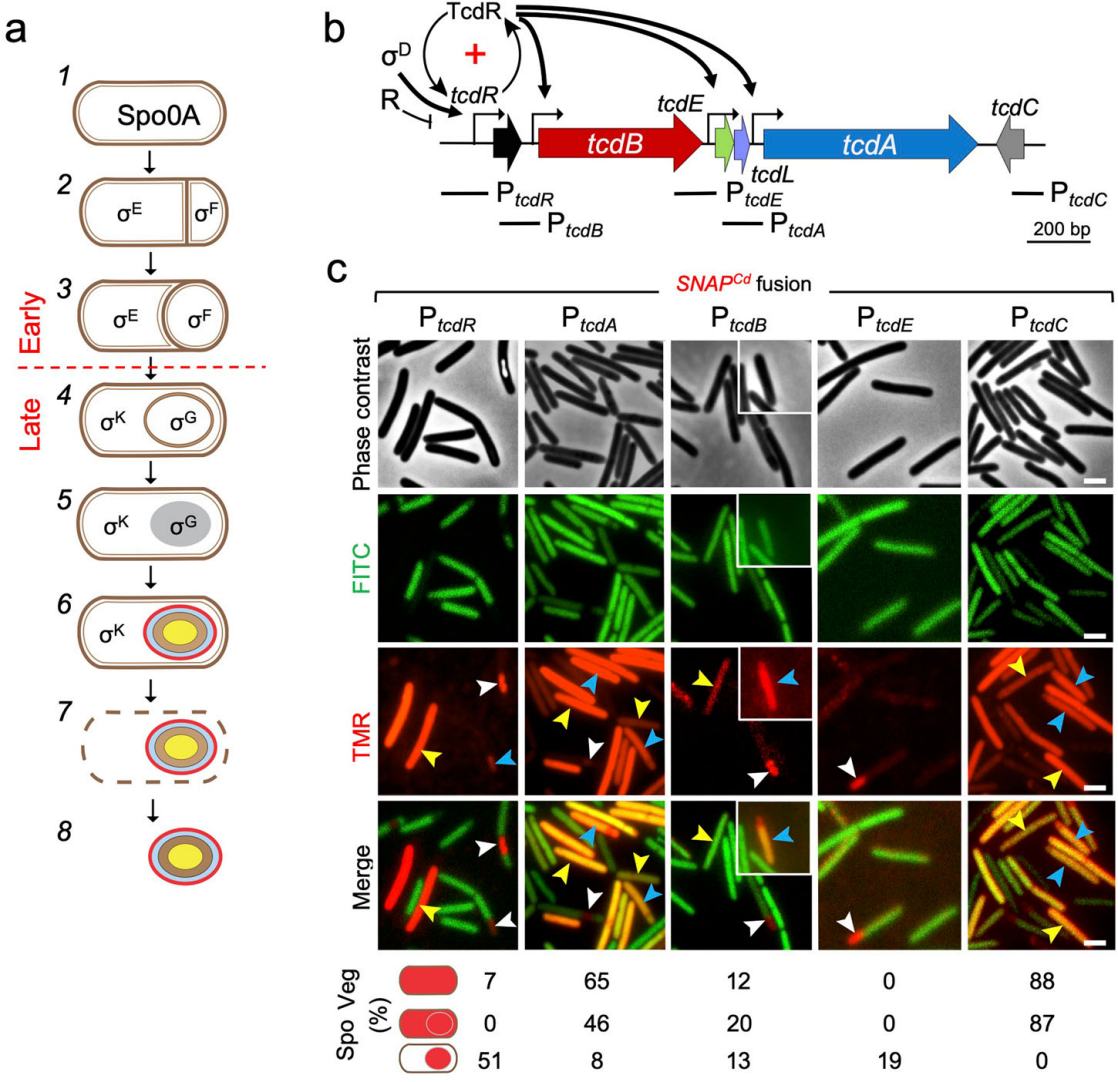
Expression of the PaLoc genes during *C. difficile* growth and sporulation. **a** Shown is the pathway of spore differentiation starting with vegetative (pre-divisional cells, (*1*), asymmetric division (*2*), a stage in engulfment (*3*), engulfment completion (*4*), synthesis of the spore protective layers (*5* and *6*) and free mature spores (*8*), resulting from mother cell lysis (*7*). Spo0A controls several stationary phase processes and is also essential for entry into sporulation. Cell type-specific gene expression results from the action of the indicated σ factors. Early and late stages in development are defined as those occurring prior to or following engulfment completion, as indicated. **b** Genetic organization of the PaLoc. Broken arrows represent promoters in the region; the TcdR positive auto-regulatory loop (“+” sign) and the role of σ^D^ in its priming are indicated. The black arrows represent direct regulation of the indicated promoters by TcdR. Other direct or indirect regulatory factors that impinge onto the expression of the PaLoc-encoded genes are collectively represented by “R”. The lines below the genetic map show the position and extent of the DNA fragments used to create the indicated transcriptional *SNAP*^*Cd*^ fusions. Note that the P_*tcdR*_ fragment includes two *tcdR*-dependent promoters (P1 and P2), the σ^D^-dependent promoter and a σ^A^-type promoter (see also Fig. 3). **c** Cell type-specific expression patterns of P_*tcdR*_*-*, P_*tcdA*_*-*, P_*tcdB*_*-*, P_*tcdE*_*-* and P_*tcdC*_*-SNAP*^*Cd*^ transcriptional fusions in strain 630Δ*erm*. The cells were collected 24 h after inoculation in TY liquid medium, labeled with TMR-Star and examined by phase contrast and fluorescence microscopy to monitor SNAP^Cd^ production. The merged images show the overlap between the TMR-Star (red) and the auto-fluorescence (green) channels. The images are representative of the expression patterns observed for the different fusions in three independent experiments. Yellow arrowheads point to vegetative cells with expression, white arrowheads point to sporulating cells with forespore-specific expression and blue arrowheads point to sporulating cells with a whole sporangium expression pattern. The various cellular patterns of SNAP^Cd^ production were scored and their percentage relative to the total number of vegetative (Veg) or sporulating cells (Spo) is shown. The images are representative of the expression patterns observed for the different fusions in three independent experiments (see also Fig. S1 and the Methods section). For sporulating cells the scoring includes a whole sporangium expression pattern and a forespore-specific pattern. The number of cells analyzed for each fusion, *n*, is as follows: P_*tcdA*_-*SNAP*^*Cd*^, *n* = 245; P_*tcdB*_-*SNAP*^*Cd*^, *n* = 410; P_*tcdC*_-*SNAP*^*Cd*^, *n* = 400; P_*tcdE*_-*SNAP*^*Cd*^, *n* = 579; P_*tcdR*_-*SNAP*^*Cd*^, *n* = 353. Scale bar, 1 μm.

Spores are formed during the stationary phase of growth. Initially, an asymmetric division partitions the rod-shaped cell into a larger mother cell and a smaller forespore, the future spore (Fig. 1a)^6,7^. Soon after division, the mother cell begins to engulf the forespore, which eventually becomes isolated from the external medium. Several protective layers are then deposited around the forespore, including a peptidoglycan layer known as the cortex essential for heat resistance, and two proteinaceous layers, the coat and a more external exosporium. These two structures contribute to spore resistance against noxious chemicals and peptidoglycan-breaking enzymes, are required for proper germination, binding to host cells and dramatically influence colonization and disease-causing ability^13–19^. Finally, lysis of the mother cell releases the spore into the environment^6,7^ (Fig. 1a). Although there are differences in the morphology of different spore layers among species, the main morphological stages of sporulation are conserved among spore-formers^6,7^.

Spo0A is a response regulator and the master regulatory protein governing entry into sporulation^20–22^. Spo0A is essential for the asymmetric division of the rod-shaped cell into a smaller forespore and a larger mother cell^23,24^ (reviewed by refs. ^6,7,9^; Fig. 1a). Once the forespore and the mother cell are formed, σ^F^ is activated in the forespore and σ^E^ is soon after activated in the mother cell. At later stages in development, σ^K^ is the main factor driving developmental regulated gene expression in the mother cell, whereas σ^G^ replaces σ^F^ in the forespore^23–25^ (Fig. 1a). While gene expression during sporulation is mainly governed by the cell type-specific sigma factors, ancillary transcription factors sub-divide the sigma regulons into several temporal and epistatic classes^20–22,26^. For example, SpoVT is produced in the forespore under the control of both σ^F^ and σ^G^, and positively regulates the expression of a subset of σ^G^-controlled genes while repressing genes under the control of σ^F^^9,25^. Importantly, the mother cell-specific σ^E^ and σ^K^ are the key players in the morphogenesis of the spore surface layers^23–25,27^.

The genes coding for TcdA and TcdB are located in a pathogenicity locus, or PaLoc^1,2,8^ (Fig. 1b). TcdA and TcdB are glucosyltransferases that belong to the family of Large Clostridial Toxins (LCTs) (reviewed in^8,28^). The toxins enter host cells via receptor-mediated endocytosis and several TcdA receptors have been proposed^8,28,29^. The PaLoc carries four other genes, *tcdR*, *tcdE*, *tcdL*, and *tcdC* (Fig. 1b). *tcdR* codes for an RNA polymerase sigma factor, TcdR, required for the transcription of *tcdA*, *tcdB* and *tcdE*^30,31^ (Fig. 1b). *tcdC* codes for a small acidic transmembrane protein thought to be a negative regulator of toxin production^32^, although its role is still unclear^33–38^. The *tcdE* gene codes for a holin-like protein; expression of *tcdE* in *Escherichia coli* complements a lambda S mutant, and under certain conditions causes cell lysis^39^. Although neither TcdA nor TcdB have recognizable secretion signals, at least in some strains TcdE appears to be required for TcdA and TcdB release^39–42^ in a process proposed to be partially redundant with stationary phase autolysis mediated by a lytic transglycosylase^43^. Finally, *tcdL* codes for a 43 amino acid-long polypeptide with structural similarity to a non-catalytic fragment of a peptidoglycan endolysin and it binds to TcdB suggesting a role in toxin transport^44,45^.

Expression of *tcdA* and *tcdB* is strongly induced early in stationary phase and persists during this phase^46,47^. Toxin production is subject to the action of several regulatory proteins and signals that converge to limit expression of the toxin-encoding genes during growth (Fig. 1b, collectively denoted as R; reviewed in ref. ^48^). The sigma factor σ^D^, in turn, a central regulator of flagellar assembly^49,50^ is a positive regulator of toxin production; it uses a promoter upstream of *tcdR* to prime a positive auto-regulatory loop involving two TcdR-dependent *tcdR* promoters^51,52^ (Fig. 1b). The TcdR auto-regulatory loop establishes a bistable switch that results in the expression of *tcdA* in only a fraction of the population^52,53^.

Some of the regulators and signals that repress toxin production also repress sporulation initiation (reviewed in ref. ^48^). For instance, the regulatory protein RstA, promotes sporulation while simultaneously acting as a repressor of toxin production, by binding to a site upstream of and overlapping the σ^D^-dependent promoter of the *tcdR* gene^54–56^. The regulatory systems that influence both sporogenesis and toxinogenesis may act mainly to bias the early stationary phase cell population towards toxin production or sporulation, in response to nutritional and other signals (^52–54,56^ reviewed in ref. ^48^). The two processes are not antithetical, however, but partially overlap as illustrated by the observation that a fraction of sporulating cells express *tcdA*^52,53^. Expression of *tcdA* was detected in the mother cell^52,53^, but it was unclear whether expression also occurred in the forespore, whether other PaLoc genes were expressed in sporulating cells, their regulation during sporulation and whether the toxins associated with spores. These are important questions, however, since binding of spores to intestinal epithelial cells, their internalization and contribution to disease recurrence is promoted by TcdA and TcdB^13,57^.

Using single-cell analysis, we show here that in a fraction of the sporulating cells, *tcdR*, *tcdA*, *tcdB* and *tcdE* exhibit forespore-specific expression, that *tcdA* and *tcdB* additionally show a whole-sporangium pattern, and that *tcdC* only shows this latter pattern. We found that expression of the PaLoc genes in the mother cell is σ^D^-dependent and most likely results from protracted σ^D^- and TcdR-dependent expression by vegetative cells. In contrast, we found that *tcdR* expression is specifically induced in the forespore under the joint control of σ^G^ and the ancillary transcription factor SpoVT. Together with SpoVT, σ^G^ utilizes a promoter in the *tcdR* regulatory region that partially overlaps the σ^D^ promoter, leading to the expression of *tcdA*, *tcdB* and *tcdE* in the forespore at a late stage in development. We demonstrate that TcdA associates with the spore surface layers and we show that wild-type spores, including those of an epidemic strain of ribotype 027, have a cytopathic effect on monolayers of HT29 and Caco-2 intestinal cells. Thus, at least TcdA is associated with mature spores in an active form and the infectious spores of *C. difficile* are toxin delivery vehicles.

## Results

### Expression of the PaLoc genes in vegetative cells

Previous work has shown that in strain 630Δ*erm* a substantial fraction of the sporangia of phase bright spores*, i.e*., at a late stage in development, expressed a P_*tcdA*_-*rfp* fusion^52^. In a more recent study, and using a dual reporter system in which *tcdA* expression was monitored using a transcriptional fusion to mNeon Green (mNG) and sporulating cells were identified using a transcriptional fusion of the σ^E^-controlled *sipL* promoter to mScarlet (mSc), the simultaneous expression of both fusions was detected in 11% of the sporulating cells^53^. While the two studies showed that sporulation and *tcdA* expression overlap to some extent, the regulation of *tcdA* expression in sporulating cells was not directly addressed, and the expression of the other PaLoc genes during sporulation was not reported^52,53^. Moreover, in the study of Ransom and co-authors, some free spores exhibited red fluorescence but whether *tcdA* was also expressed in the forespore or whether the reporter, produced in the mother cell, associated with the developing spore was unclear^52^. Both studies found that the auto-fluorescent proteins used were not sensitive forespore-specific reporters^52,53^.

Here, we have constructed derivatives of strain 630Δ*erm* bearing transcriptional fusions of the *tcdA*, *tcdB*, *tcdC*, *tcdE* and *tcdR* promoter regions to the SNAP^*Cd*^ reporter^58,59^ and used phase contrast and fluorescence microscopy to monitor the expression of the PaLoc genes across the cell population during sporulation. Note that no promoter has been identified between the *tcdE* and *tcdL* genes^44,45^. Also of note, the *tcdA* promoter fragment used for the construction of the SNAP^*Cd*^ transcriptional fusion is similar to that used in the two studies mentioned above^52,53^ (Fig. 1b; see also the Supplementary Information). The P_*tcdR*_-*SNAP*^*Cd*^ transcriptional fusion is termed full-length to distinguish it from a shorter version, described in a section below.

Samples were collected from cultures of strains bearing the various fusions 24 h after inoculation in TY, a medium that supports both sporulation and toxin production^46,52,53^. The cells were labeled with the red-fluorescent SNAP substrate TMR-Star and processed for phase contrast and fluorescence microscopy. Sporulation was evaluated by both the accumulation of partially or fully phase bright spores and by the pattern of green auto-fluorescence characteristic of *C. difficile*^60^; auto-fluorescence allows division septa and the forespore, which shows reduced auto-fluorescence relative to the mother cell, to be identified (^27,60^; Supplementary Fig. S1a). For reference, we also scored expression of the PaLoc genes in vegetative cells^52,53^. Expression of P_*tcdA*_*-SNAP*^*Cd*^ was detected in 65% of the vegetative cells (TcdA-ON cells; Fig. 1c, yellow arrowheads) while the remaining cells showed no signal. This bimodal pattern of P_*tcdA*_*-SNAP*^*Cd*^ expression is consistent with earlier results: P_*tcdA*_*-rfp* expression was detected in 85% of the vegetative cells in the study of Ransom and co-authors^52^, and in 61% or 37% of the cells as assessed with the mNG or mSc reporters, respectively, by Donnelly and co-authors^53^. Transcription of *tcdR* or *tcdB* was not detected using the RFP reporter in 630Δ*erm*; in a congenic Δ*codY* mutant, however, *tcdB* expression was detected and was bimodal^52^. In the present study, expression of P_*tcdB*_*-SNAP*^*Cd*^ was detected in 12% of the vegetative cells of the 630Δ*erm* and was thus bimodal (Fig. 1c, yellow arrowheads). Previous work has shown that *tcdB* has around 10-100-fold lower expression levels when compared with *tcdA*^33,61,62^. It thus seems possible that the lower fraction of TcdB-ON cells, relative to the fraction of TcdA-ON cells, is because the signal from P_*tcdB*_-*SNAP*^*Cd*^ is, in a fraction of cells, too close to the background to be detected. Expression of P_*tcdR*_-SNAP^Cd^ itself, was only detected in a sub-population of about 7% of the vegetative cells (Fig. 1c, yellow arrowheads). The lower fraction of cells may again reflect our detection limit and/or the rate of transcription initiation from the *tcdR* promoter, which is lower than from the *tcdA* or *tcdB* promoters^46^.

The bimodal pattern of *tcdA* expression has its origin in the TcdR auto-regulatory loop and one prediction was that the expression of *tcdB* and of *tcdR* itself could also be bimodal^52^. Here, we confirm this expectation, in that as for *tcdA*, only a fraction of the cells are in a TcdB-ON or TcdR-ON state. In contrast, we did not detect expression of *tcdE* in vegetative cells (Fig. 1c). Finally, *tcdC* expression was detected in 88% of the vegetative cells; this gene, however, is not known to be under TcdR control (Fig. 1c, yellow arrowheads). In all cases, complete labeling of the SNAP^Cd^ reporter was achieved (Supplementary Figs. S2 and S9). Although there was some variation in the percentage of ON/OFF cells between experiments, the general pattern of bimodal transcription, verified for the P_*tcdR*_ and P_*tcdA*_ promoters is maintained (7 ± 3% for TcdR-ON cells; 60 ± 12% for TcdA-ON cells, as assessed in 8 independent experiments; Supplementary Fig. S1b).

### Expression of the PaLoc genes in sporulating cells

We then examined the expression of the PaLoc genes during sporulation. Control experiments showed very similar sporulation kinetics and efficiencies (all below 10%) and kinetics for the strains bearing the various transcriptional fusions as measured 12, 24, and 48 h after inoculation (Supplementary Fig. S1c). The exception was the P_*tcdR*_*-SNAP*^*Cd*^-bearing strains, which showed an efficiency of sporulation below 10% at hour 12 but greater than 50% at hours 24 and 48 (Supplementary Fig. S1c). The reason for this behavior is not presently understood but one possibility is that the DNA fragment fused to the *SNAP*^*Cd*^ reporter contains a site that titrates out a negative regulator of sporulation. In any event, it seems unlikely that the increased sporulation efficiency of the P_*tcdR*_*-SNAP*^*Cd*^*-*bearing strain introduces a bias in our scoring of the fraction of *tcdR*-ON cells.

During sporulation, expression of *tcdR* was detected in 51% of the sporulating cells, specifically in the forespore (Fig. 1c, white arrowheads). The forespore-specific expression of *tcdR* was mainly seen in sporangia that had completed the engulfment process and thus, at a late stage in development (Fig. 1c). Expression of *tcdA* was also detected in sporulating cells, but with a more complex pattern than that found for *tcdR*; while expression was confined to the forespore in 8% of the sporangia scored (Fig. 1c, white arrowheads), 46% showed a whole sporangia pattern, i.e., expression in both the forespore and the mother cell (Fig. 1c, blue arrowheads). Importantly, we did not detect expression of *tcdA* only in the mother cell; in the study of Ransom and co-authors, what seems to be mother cell-specific *tcdA* expression may result from the low sensitivity of the RFP reporter in the forespore (see also above^52^;). SNAP^Cd^, however, is an efficient reporter for gene expression in the forespore^24,25,27,58^ .

As for *tcdA*, expression of *tcdB* was also detected in sporulating cells; of these, 13% showed expression only in the forespore (Fig. 1c, white arrowheads), whereas 20% showed a whole sporangium pattern (blue arrowhead). Contrasting with the vegetative cells, expression of *tcdE* was only detected, albeit weakly, in sporulating cells and only in the forespore (19% of the sporangia scored; Fig. 1c, white arrowheads). Finally, 87% of the sporulating cells showed a whole sporangia pattern of P_*tcdC*_-*SNAP*^*Cd*^ expression (Fig. 1c, blue arrowheads), But unlike the other PaLoc genes, expression of *tcdC* was not detected in the forespore only. As also shown for the expression in vegetative cells (above), there was some variation between experiments in the percentage of cells showing P_*tcdR-*_ and P_*tcdA*_-*SNAP*^*Cd*^ expression in whole-sporangia (52 ± 10% for *tcdA*, less that 3% for *tcdR*) or the forespore (50 ± 10% for *tcdR*; 5 ± 3% for *tcdA*) (Supplementary Fig. S1b).

In summary, in line with earlier work^52^, we detected expression of *tcdA* in sporulating cells. Moreover, we found that *tcdR*, *tcdA*, *tcdB* and *tcdE* exhibit forespore-specific expression, while *tcdA* and *tcdB* additionally show a whole-sporangium pattern, and *tcdC* only shows this latter pattern. Regardless of the pattern, forespore or whole-sporangium, the bimodality in expression of the PaLoc genes seen in vegetative cells is also observed in sporulating cells.

### Expression of the PaLoc genes during sporulation is TcdR-dependent

TcdR is required for expression of the PaLoc genes *tcdA*, *tcdB*, and *tcdE*^30,31,63^. To determine whether TcdR was required for the expression of the PaLoc genes during sporulation, we first constructed a *tcdR* in-frame deletion mutant using Allelic Coupled Exchange^64^. The insertional inactivation of *tcdR* in strain 630Δ*erm* was reported to cause a small, two-fold increase in sporulation^65^, a result that we also obtained (Supplementary Table 1).

**Fig. 2:**
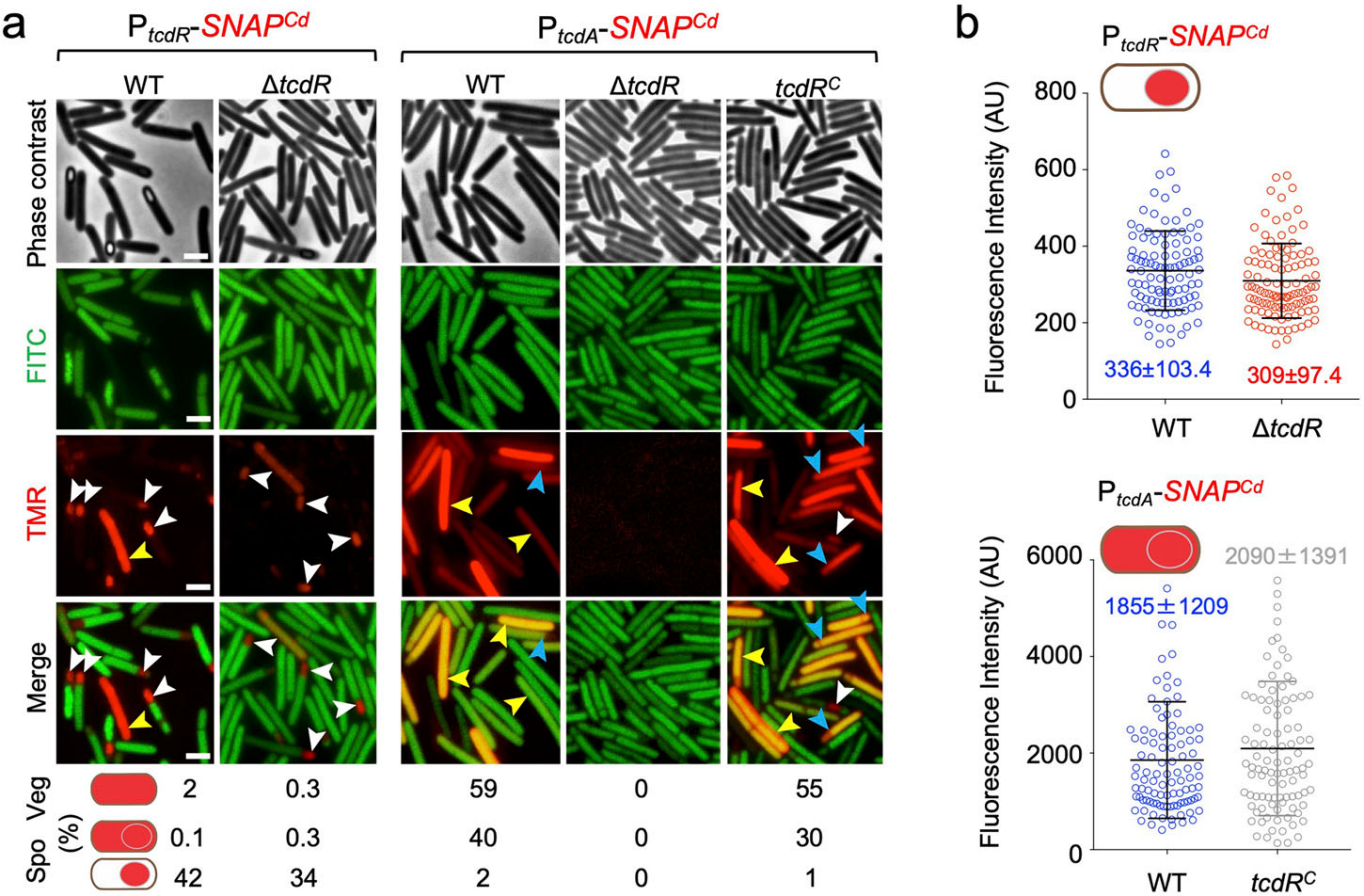
The role of TcdR in regulating toxin production. **a** Microscopy analysis of *C. difficile* cells carrying transcriptional fusions of the *tcdR* and *tcdA* promoters to *SNAP*^*Cd*^ in strain 630Δ*erm* (WT), in the Δ*tcdR* mutant and in the Δ*tcdR* mutant complemented with the wild-type allele at the *pyrE* locus (*tcdR*^*C*^). The cells were collected after 24 h of growth in TY liquid medium, labeled with TMR-Star and examined by phase contrast and fluorescence microscopy to monitor SNAP production. The merged images show the overlap between the TMR-Star (red) and the auto-fluorescence (green) channels. The images are representative of the expression patterns observed for the different fusions in three independent experiments (see also Fig. S1b and the Methods section). Yellow arrowheads point to vegetative cells with expression, white arrowheads point to sporulating cells with forespore-specific expression and blue arrowheads point to sporulating cells with whole sporangium SNAP^Cd^ production. The numbers below the panels show the percentage of vegetative (Veg) cells and sporulating cells (Spo) with the represented patterns. The number of cells analyzed for each strain, *n*, is as follows: WT with P_*tcdA*_-*SNAP*^*Cd*^, *n* = 918; *tcdR*^*C*^ with P_*tcdA*_-*SNAP*^*Cd*^, *n* = 820; WT with P_*tcdR*_-*SNAP*^*Cd*^, *n* = 1456; Δ*tcdR* with P_*tcdR*_-*SNAP*^*Cd*^, *n* = 2246; *tcdR*^*C*^ with P_*tcdR*_-*SNAP*^*Cd*^, *n* = 2688. Scale bar, 1 μm. **b** Quantitative analysis of the fluorescence intensity (in Arbitrary Units, AU) of the SNAP^Cd^ signal per forespore for the *tcdR* fusion and in whole sporangia for the *tcdA* fusion, in the WT or in the Δ*tcdR* and *tcdR*^*C*^ strains; the data refers to the experiments described in (**a**). The numbers inside the graphs represent the mean value ± the standard deviation. All pairwise comparisons were non-significant (see Methods).

We then examined expression of P_*tcdR-*_ and P_*tcdA*_-*SNAP*^*Cd*^ during sporulation in a Δ*tcdR* mutant, the latter as a proxy for the expression of the toxin-encoding genes. Surprisingly, expression of P_*tcdR*_*-SNAP*^*Cd*^ during sporulation, was still detected in the Δ*tcdR* mutant, both in whole sporangia (Fig. 2a, 0.3%) and mostly in the forespore (white arrowheads; 34% of the sporangia scored). Moreover, the average intensity of the fluorescence signal in the forespore (Fig. 2b; 309 ± 97.4 AU) did not differ significantly from the WT (Fig. 2b; 336 ± 103.4 AU). Thus, TcdR is not required for the forespore-specific expression of *tcdR*. In sharp contrast, expression of P_*tcdA*_*-SNAP*^*Cd*^ was not detected in sporulating cells of the Δ*tcdR* mutant but was restored when a *tcdR* was inserted at the *pyrE* locus in single copy (Fig. 2, *tcdR*^*C*^ strain). Complementation could be observed for the whole sporangia pattern, both in the percentage of cells (Fig. 2a; 40% in the WT, 30% in the *tcdR*^*C*^ strain), as well as in the average intensity of the fluorescence signal from P_*tcdA*_*-SNAP*^*Cd*^ (Fig. 2b; 1855 ± 1209 AU in the WT, 2090 ± 1391 in the *tcdR*^*C*^ strain).

Since *tcdR* expression in sporulating cells does not require TcdR whereas *tcdA* expression is *tcdR-*dependent, we infer that a factor other than TcdR most likely drives *tcdR* expression in sporulating cells.

### Expression of the PaLoc genes in whole sporangia, but not in the forespore, is σ^D^-dependent

**Fig. 3:**
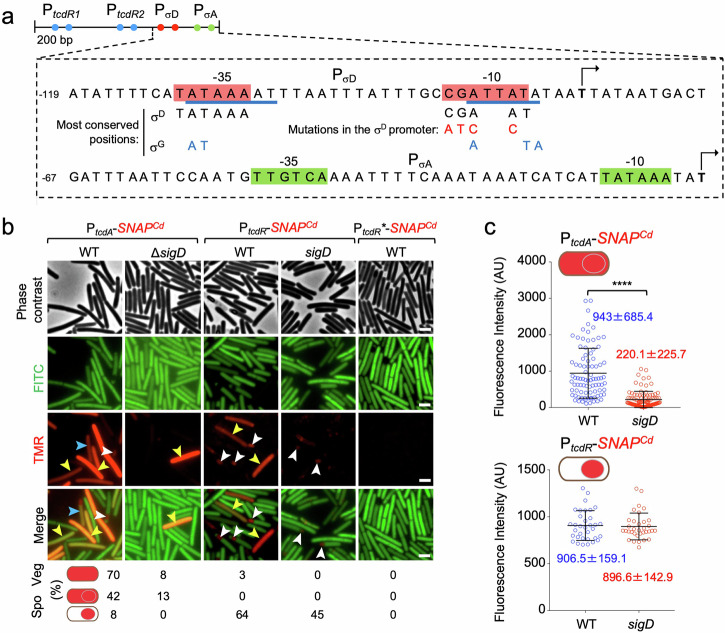
σ^D^ regulates toxin production in whole sporangia but not in the forespore. **a** Shows the regulatory region of the *tcdR* gene, with the −35 and −10 elements of the tandem *tcdR*-dependent promoters (P_*tcdR1*_ and P_*tcdR2*_, blue dots) and the σ^D^- and σ^A^-dependent promoters (red and green dots). The bases underlined in blue indicate a putative σ^G^-dependent promoter. The most conserved positions for σ^D^ and σ^G^-dependent promoters are shown as well as the point mutations introduced in the −10 region of the σ^D^ promoter (letters in red). Transcriptional start sites are indicated by broken arrows. Numbering is relative to the start site downstream of the σ^A^-type promoter. **b** Microscopy analysis of *C. difficile* cells carrying fusions of the *tcdA, tcdR* and tcdR* (with point mutations in the σ^D^-dependent promoter) promoters to *SNAP*^*Cd*^ in strain 630Δ*erm* (WT) and in the Δ*sigD* mutant. The cells were collected after 24 h of growth in TY liquid medium, labeled with TMR-Star and examined by phase contrast and fluorescence microscopy to monitor SNAP^Cd^ production. The merged images show the overlap between the TMR-Star (red) and the auto-fluorescence (green) channels. The images are representative of the expression patterns observed for the different fusions in three independent experiments (see Methods). Yellow arrowheads point to vegetative cells with SNAP^Cd^ expression, white arrowheads point to sporulating cells with forespore-specific expression and blue arrowheads point to sporulating cells with whole sporangium expression. To score the indicated patterns in vegetative (Veg) or sporulating cells (Spo) the number of cells analyzed for each strain, *n*, was as follows: WT with P_*tcdA*_-*SNAP*^*Cd*^, *n* = 856; *sigD* with P_*tcdA*_-*SNAP*^*Cd*^, *n* = 1550; WT with P_*tcdR*_-*SNAP*^*Cd*^, *n* = 670; *sigD* with P_*tcdR*_-*SNAP*^*Cd*^, *n* = 3902; WT with P_tcdR*_-*SNAP*^*Cd*^, *n* = 268. **c** Fluorescence intensity (in Arbitrary Units, AU) of the SNAP^Cd^ signal per sporangia for the *tcdA* fusion and in the forespore for the *tcdR* fusion, in the WT or in the Δ*sigD* mutant. The numbers in the panels represent the mean value ± the standard deviation. ****, *p* < 0.0001; no stars, non-significant differences (see Methods). Scale bar, 1 μm.

A σ^D^-dependent promoter was previously mapped upstream of *tcdR*^49,51,66^ (Fig. 3a). To explore the relevance of this promoter for *tcdR* expression in the forespore we fused a fragment from the *tcdR* regulatory region containing the σ^D^ promoter to the *SNAP*^*Cd*^ reporter; this transcriptional fusion is called P_*tcdR-D*_-*SNAP*^*Cd*^ (Supplementary Fig. S3a). In sporulating cells, no whole-sporangium pattern was detected using P_*tcdR-D*_ (Supplementary Fig. S3b). The percentage of forespores showing P_*tcdR-D*_ expression (44%), however, did not differ significantly from that obtained with the full-length fusion (52%; Supplementary Fig. S3b). Moreover, the intensity of the fluorescence signal in the forespore also did not differ significantly between the two fusions (Supplementary Fig. S3c). Together, these results indicate that the region containing the σ^D^ promoter is sufficient for expression of *tcdR* in the forespore. σ^D^, however, is not known to be directly involved in sporulation^48^, leading us to hypothesize that other, sporulation-specific factor could recognize sequences within the P_*tcdR-D*_ fragment.

Although our P_*tcdR-D*_ fusion includes the σ^A^-type promoter located just downstream of the σ^D^ promoter (Supplementary Fig. S3a), the former shows weak activity (^56^; see also below). To test whether the σ^D^ promoter was involved in the forespore-specific expression of *tcdR*, we introduced point mutations in the -10 region of the promoter in the context of the full length of P_*tcdR*_*-SNAP*^*Cd*^ fusion. The most conserved positions in the σ^D^ promoter^51,67^ are shown in Fig. 3a, together with the substitutions introduced in the -10 region of the promoter. The new fusion, carrying the mutations in the -10 region of the σ^D^ promoter was designated P_tcdR*-_*SNAP*^*Cd*^ (Fig. 3a). Additionally, we introduced the P_*tcdR*_- and P_*tcdA*_*-SNAP*^*Cd*^ fusions into a *sigD* mutant^51^.

Single-cell analysis showed that in a *sigD* mutant, the fraction of vegetative cells expressing *tcdA* was reduced from 70% to 8% (Fig. 3b, yellow arrowheads). Moreover, TcdR-ON cells were not detected. This is consistent with the role of σ^D^ in activating the TcdR auto-regulatory loop^52^.

In sporulating cells, the whole-sporangia pattern of P_*tcdA-*_*SNAP*^*Cd*^ was reduced from 42% to 13% (Fig. 3b, blue arrowheads) and the average intensity of the signal measured in the mother cell decreased significantly relative to the WT (Fig. 3c; from 943 ± 685.4 AU to 220.1 ± 225.7 AU in the *sigD* mutant). Importantly, the forespore-specific expression of *tcdR* was still detected in 45% of the forespores scored, as compared to 65% for the WT (Fig. 3b, white arrowheads), and the average intensity of the fluorescence signal per forespore did not differ significantly from the WT (Fig. 3c; 906.5 ± 159.1 AU for the WT and 896.6 ± 142.9 AU for the *sigD* mutant).

The results suggest that the whole sporangia pattern of expression results mostly from either persistent activity of σ^D^ in the mother cell and the forespore following asymmetric division, or that TcdR, produced under σ^D^ control in pre-divisional cells partitions between the two cells following asymmetric division.

The point mutations introduced in the −10 region of the σ^D^ promoter, abolished expression of P_tcdR*-_*SNAP*^*Cd*^ in vegetative cells, again consistent with the role of σ^D^ in driving production of TcdR and arguing in favor of a minor role for the σ^A^-type promoter in *tcdR* expression (Fig. 3b). Strikingly, however, the forespore-specific expression of P_tcdR*-_*SNAP*^*Cd*^ was also abolished (Fig. 3b). Since expression of P_*tcdR-*_*SNAP*^*Cd*^ in the forespore is not affected by deletion of *sigD* (above), we infer that the point mutations affect sequences that are also recognized by a forespore-specific regulatory factor other than σ^D^. The target for this factor must at least partially overlap the σ^D^-dependent promoter.

### Expression of the PaLoc genes in the forespore is under σ^G^ control

Having established that the region required for forespore-specific expression of *tcdR* overlaps the σ^D^-recognized promoter, we next aimed at identifying the forespore-specific factors involved. We started by evaluating *tcdR* expression in *sigF*, sigE, *sigG* and *sigK* mutants, all of which are unable to sporulate, and in *spo0A* cells, for reference. In the *spo0A* mutant, vegetative expression of P_*tcdR-*_*SNAP*^*Cd*^ is maintained, although the number of cells expressing *tcdR* was reduced to 0.6% as compared to 7% for the WT (Supplementary Fig. S4). Thus, under our conditions, Spo0A seems to positively control toxin production, at least in a fraction of vegetative cells, consistent with previous reports^53,68–72^. Unlike the pleiotropic *spo0A* mutation, deletion of *sigF* only affects sporulation. In the *sigF* mutant, which is arrested in development just after asymmetric division^23,24^, P_*tcdR-*_*SNAP*^*Cd*^ expression was not detected in either the mother cell and/or the forespore (Supplementary Fig. S4). The absence of vegetative cells expressing *tcdR* in the *sigF* mutant (7% in the WT; Supplementary Fig. S4) seems to suggest that a contribution from cells outgrowing from germinated spores is lacking (see also below). Importantly, no forespore-specific expression of P_*tcdR-*_*SNAP*^*Cd*^ was observed in a *sigF* mutant, indicating the involvement of σ^F^ or a σ^F^-dependent factor (Supplementary Fig. S4). Since *tcdR* expression is mostly detected following engulfment completion (above), we anticipated that the late σ^G^ factor could be required for the forespore-specific expression of *tcdR*. Consistent with this inference, in a *sigG* mutant, which completes the engulfment process but does not proceed further into development^24^, expression of *tcdR* was only detected in 28% of the forespores, as compared with 49% for the WT (Supplementary Fig. S4). We presume that expression of *tcdR* is reduced but not abolished in the *sigG* mutant because under certain conditions σ^F^ can utilize promoters that are normally recognized by σ^G^, the two sigma factors having very similar recognition sequences^23,24,67^. In cells unable to produce the early mother cell regulator σ^E^, *tcdR* expression was reduced to 9% of the sporangia (Supplementary Fig. S4) consistent with a requirement for σ^E^ for the full activity of σ^G^^24,25,27,73^. In cells unable to produce the late, mother cell-specific regulatory protein σ^K^, the forespore-specific expression of *tcdR* was reduced (to 29% of the sporangia, similar to the *sigG* mutant; Supplementary Fig. S4). This suggests that σ^K^ influences late gene expression in the forespore (see also the Discussion).

The sequences for promoter recognition by σ^G^ are very similar in *B. subtilis* and *C. difficile*, and include the −35 and −10 elements, GAATAAAAT and ATAATA, with a spacing of 15 bp^25^. While sequences that approach the consensus for σ^G^-recognized -10 element overlap the -10 region of the σ^D^ promoter upstream of *tcdR*, the −35 element is less conserved (Fig. 3a, nucleotides underlined in blue; the most conserved bases are also indicated). Therefore, σ^G^ may drive the post-engulfment expression of *tcdR* in the forespore by recognizing sequences that partially overlap the σ^D^ promoter.

### σ^G^ and SpoVT control expression of the PaLoc genes in the forespore

**Fig. 4:**
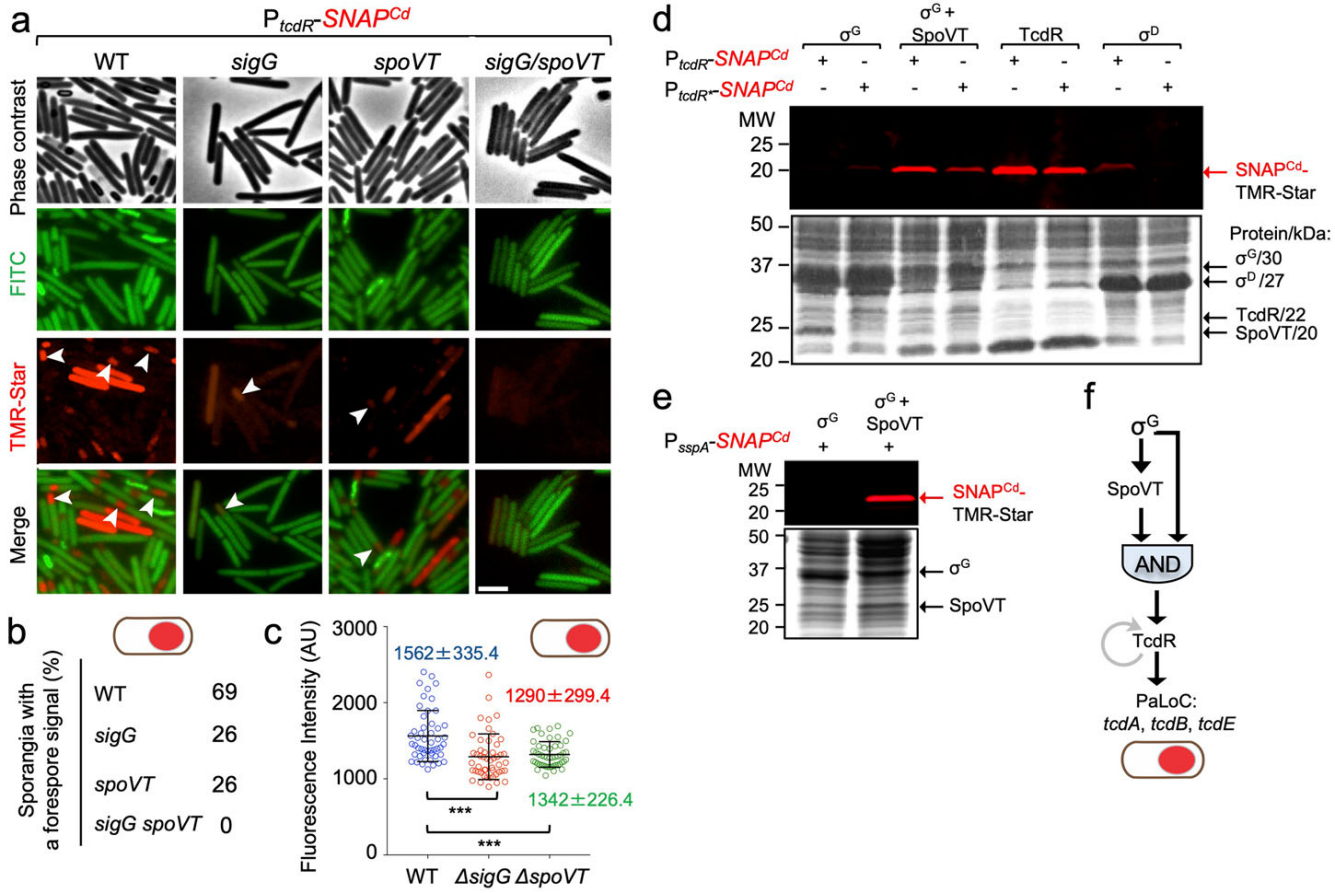
Toxin production in the forespore is dependent on σ^G^ and SpoVT. **a** Strains of *C. difficile* carrying a fusion of the *tcdR* regulatory region to *SNAP*^*Cd*^ in strain 630Δ*erm* (WT) and in the *sigG*, *spoVT* and *sigG*/*spoVT* were grown in TY medium and cells collected after 24 h of growth. The cells were labeled with TMR-Star and examined by phase contrast and fluorescence microscopy. The merged images show the overlap between the TMR-Star (red) and the auto-fluorescence (green) channels. The images are representative of the P_*tcdR*_-*SNAP*^*Cd*^ expression patterns seen in three independent experiments, with white arrowheads identifying forespore-specific expression. Scale bar, 1 μm. **b** Shows the percentage of sporangia with a forespore-specific signal for the indicated strains. The number of cells analyzed for each strain, *n*, was: WT, *n* = 450; *sigG*, *n* = 878; *spoVT*, *n* = 540; *sigG*/*spoVT*, *n* = 500. **c** Fluorescence intensity (in Arbitrary Units, AU) of the SNAP^Cd^ signal in the forespore for the P_*tcdR*_*-SNAP*^*Cd*^ fusion in the WT or congenic *sigG* and *spoVT* mutants. Note that no signal was detected for the *sigG*/*spoVT* double mutant. The numbers in the panels represent the mean value ± the standard deviation. ***, *p* < 0.001 (see Methods); **d** *E. coli* BL21(DE3) derivatives with plasmids carrying P_*tcdR*_- or P_tcdR*_-*SNAP*^*Cd*^ fusions, as indicated, were transformed with compatible plasmids for the induction of σ^G^, σ^G^, and SpoVT, TcdR or σ^D^. **e** As in (**d**), except that *E. coli* BL21(DE3) containing a plasmid with a P_*sspA*_*-SNAP*^*Cd*^ fusion was transformed with plasmids for the induction of σ^G^ alone or together with SpoVT, as indicated. In (**d**) and (**e**), the various proteins were produced through auto-induction, whole cell extracts prepared, labeled with TMR-Star, proteins resolved by SDS-PAGE, and the gels scanned on a fluoroimager (top) before Coomassie staining (bottom). The position of the various regulatory proteins is indicated by black arrows and their molecular weights are given on the right side of (**d**). The position of the SNAP^Cd^-TMR-Star complex is indicated by the red arrows. The regions shown in panels d and e were cropped from original gels shown in Supplementary Fig. S10. **f** Representation of the coherent feed-forward loop formed by σ^G^ and SpoVT, with an AND gate logic, though to lead to delayed production of TcdR in the forespore, and to the expression of the PaLoc genes *tcdA*, *tcdB*, *tcdR*, and *tcdE* in this cell.

The results suggest the involvement of σ^G^ in the forespore-specific expression of *tcdR*, and a possible σ^G^-dependent promoter overlaps the σ^D^ -recognized promoter (Fig. 3a and Supplementary Fig. S3a). Since the expression of a subset of σ^G^-dependent genes additionally requires SpoVT^9,25^ we wanted to monitor expression of *tcdR* in a *spoVT* mutant, and in a *sigG*/*spoVT* double mutant. Single-cell analysis revealed forespore-specific expression of *tcdR* in 26% of the *spoVT* sporangia scored (Fig. 4a, white arrowheads), similar to the fraction seen for the *sigG* mutant but lower than for the WT (69%; Fig. 4b).

Importantly, in the *sigG*/*spoVT* double mutant no signal for *tcdR* expression could be detected (Fig. 4b). Moreover, the average intensity of the fluorescence signal per forespore in the *spoVT* mutant (1342 ± 226.4 AU) was lower than in the WT (1562 ± 335.4 AU) and similar to the *sigG* mutant (1290 ± 299.4 AU) (Fig. 4c). These results support the idea that SpoVT, together with σ^G^, is involved in regulating the expression of *tcdR* in the forespore.

To test whether σ^G^ could, together with SpoVT, directly utilize a promoter in the *tcdR* regulatory region, σ^G^ was overproduced alone or in combination with SpoVT, from the IPTG-inducible T7*lac* promoter, in *E. coli* cells bearing a compatible plasmid carrying either the wild-type P_*tcdR*_-*SNAP*^*Cd*^ fusion or the version with the point mutations in the −10 region of the σ^G^ promoter, P_tcdR*_-*SNAP*^*Cd*^. Additionally, expression of the fusions was also monitored in *E. coli* cells overproducing either TcdR or σ^D^ (Fig. 4d; see also Supplementary Fig. S10a). Accumulation of SNAP^Cd^ was used as a proxy for the utilization of the WT or mutant forms of the *tcdR* promoter by the various regulatory proteins. As expected, induction of *tcdR* resulted in high-level production of SNAP^Cd^ (Fig. 4d). Also as expected, induction of *sigD* expression led to SNAP^Cd^ production from the WT but not from the P_tcdR*_ mutant promoter (Fig. 4d). When *sigG* only was induced, SNAP^Cd^ was not detected from neither the WT nor the mutant promoter. Strikingly, however, when *sigG* and *spoVT* were co-expressed, SNAP^Cd^ production was detected from the WT P_*tcdR*_ promoter, but at much lower levels from the mutant promoter, P_tcdR*_ (Fig. 4d). In a control for the *E. coli* induction assay, we used a P_*sspA*_-*SNAP*^*Cd*^ fusion, as expression of *sspA* is known to be under the joint control of σ^G^ and SpoVT^23,25^. The P_*sspA*_-driven production of SNAP^Cd^ required the induction of both σ^G^ and SpoVT (Fig. 4e and Supplementary Fig. S10b). Thus, our in vitro assay suggests that together with SpoVT, σ^G^ is able to induce P_*tcdR*_-SNAP^Cd^ expression by recognizing a promoter that overlaps the one utilized by σ^D^ in vegetative cells. σ^G^ and SpoVT thus establish a coherent feed forward loop that controls *tcdR* expression in the forespore (Fig. 4f).

### TcdA associates with the spore surface

**Fig. 5:**
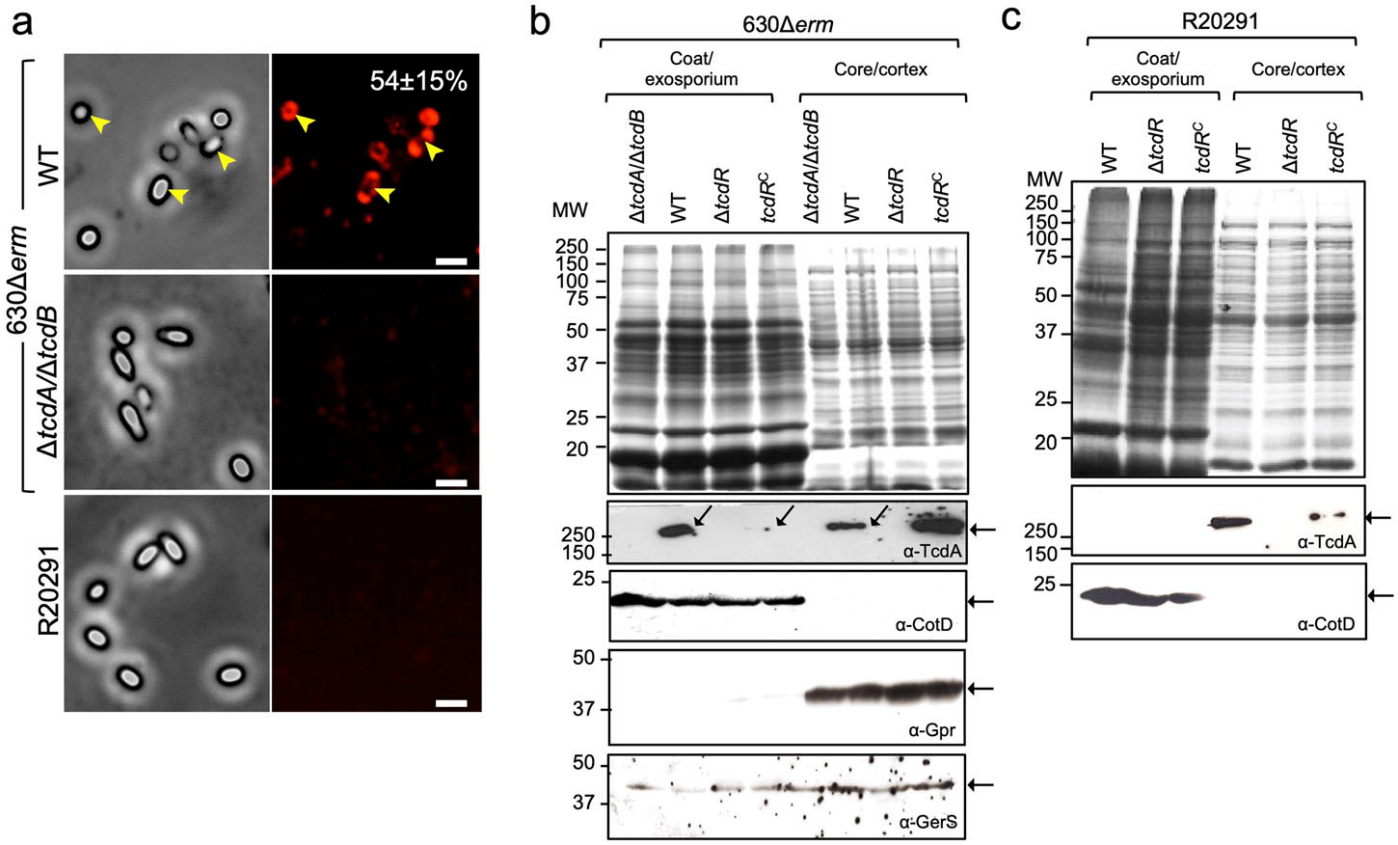
TcdA localization in mature spores. **a** The accessibility of TcdA to an anti-TcdA antibody was examined in spores of strains 630Δ*erm*, Δ*tcdA/*Δ*tcdB* and R20291 by immunofluorescence. The numbers in the top right image represent the percentage of 630Δ*erm* spores with a fluorescence signal, indicated by yellow arrowheads (average of three independent experiments ± standard deviation). Fractionation of 630Δ*erm* (**b**) or R20191 (**c**) spores, their congenic Δ*tcdA*Δ*tcdB* and Δ*tcdR* derivatives and a Δ*tcdR* mutant complemented with the wild-type allele at the *pyrE* locus, *tcdR*^*C*^. Intact mature spores were fractionated into a coat/exosporium and a core/cortex fraction. The proteins in the various fractions were resolved by SDS-PAGE (top) and subject to immunoblotting (bottom) with anti-TcdA, and anti-CotD antibodies (CotD is a bona fide coat protein). For 630Δ*erm* spores, the extracts were also probed with anti-GPR (GPR is a germination protease localized in the spore core) and anti-GerS antibodies (GerS is a cortex-modifying protein involved in spore germination). The regions shown in panels b and c were cropped from the original immunoblots shown in Supplementary Figs. S11 and S12.

Since, with the exception of *tcdC*, expression of the PaLoc genes was detected in sporulating cells, we wanted to test whether the toxins associated with mature spores. *C. difficile* spores were purified and utilized in immunofluorescence assays using an anti-TcdA monoclonal antibody; the presence of TcdB in spores was not assessed as we do not have an antibody with sufficient specificity and/or sensitivity. We found that an anti-TcdA antibody strongly decorated a fraction of the purified WT spores (Fig. 5a; 54 ± 15%) but not those of a Δ*tcdA/*Δ*tcdB* double mutant. The accessibility of TcdA to the anti-TcdA antibody in spores of strain 630Δ*erm* suggests that the toxin is associated with the coat and/or exosporium, the outermost layers of spores; protein components of these structures have been detected by immunofluorescence^18,19,74^. In the epidemic ribotype 027, strain R20291, however, spore-associated TcdA was not detected by immunofluorescence, suggesting that the toxin either does not associate with the spore or is not accessible to the antibody (Fig. 5a).

### TcdA associates with different spore layers

To independently test the association of TcdA with spores and its localization within spores, we used biochemical fractionation. Spores formed by strains 630Δ*erm* and R20291 and spores formed by their Δ*tcdR* derivatives were boiled in the presence of SDS and DTT to extract proteins present in the coat and exosporium layers^18,24,73–77^. These proteins define a coat/exosporium fraction (Fig. 5b, c; see also Supplementary Figs. S11, S12).

An earlier study reported that the insertional inactivation of *tcdR* in strain R20291 resulted in a three-fold reduction in spore heat resistance^65^. Under our culturing conditions, however, the in-frame deletion of *tcdR* in R20291 or 630Δ*erm*, caused a small, three-fold increase in spore heat resistance (Table S1). Spores of all strains remained phase bright after extraction, indicating that the cortex and core did not suffer major structural alterations^24^.

Following extraction of the coat and exosporium, the spores were treated with lysozyme, leading to at least the partial release of proteins that are in proximity to or within the cortex peptidoglycan^78^, and most likely also of spore core proteins. The lysozyme digestion defined a core/cortex fraction (Fig. 5b, c). Proteins in the two fractions were then resolved by SDS-PAGE and the gels stained with Coomassie (Fig. 5b, c, top panels; loading control) and subject to immunoblotting analysis with the anti-TcdA antibody. TcdA was detected in both the coat/ exosporium and core/cortex fractions of 630Δ*erm* spores, although to a higher level in the coat/ exosporium (Fig. 5b, middle panel). We then extended this analysis to the fractions obtained from R20291 spores. We detected TcdA in the core/cortex fraction but not in the coat/ exosporium fraction (Fig. 5c; middle panel; see also Supplementary Fig. S12). Importantly, for both 630Δ*erm* and R20291 spores, TcdA was not detected in the fractions prepared from Δ*tcdR* spores, but complementation with the wild-type allele at the *pyrE* locus partially restored its association with the coat/exosporium and core/cortex for 630Δ*erm* (Fig. 5b) and with the core/cortex fraction for R20291 spores (Fig. 5c).

We used several antibodies to control for the fraccionation procedure using 630Δ*erm* spores. CotD was only detected in the coat/ exosporium fraction, consistent with its reported association with both the coat and exosporium structures^74,77^. On the other hand, GerS, required for cortex modification during spore germination^79^, could be detected in both fractions (Fig. 5b, bottom panel). It seems possible that GerS associates with both the coat/exosporium and the cortex, but that during the initial steps of germination it re-localizes exclusively to the cortex^79^. Finally, GPR, a known core protein^23–25,80^, could only be detected in the lysozyme-treated fraction, suggesting that this fraction indeed includes core-associated proteins (Fig. 5b, bottom panel; see also Supplementary Fig. S11). Overall, this analysis suggests that our fractionation procedure enriched for coat/exosporium proteins such as CotD, which are not detected in the core/cortex fraction (Fig. 5b, c).

Together, these results do not exclude the presence of TcdA in the spore core, but they indicate that TcdA associates with the coat/exosporium layers and is accessible to antibodies in 630Δ*erm* spores, whereas in spores of the epidemic strain R20291, TcdA has a more internal localization, within the core and/or cortex, or the epitope recognized by the anti-TcdA antibody is not exposed in the coat/exosporium.

To estimate the total amount of toxin extractable from spores, spores formed by strains 630Δ*erm* and R20291 were biochemically fractionated and TcdA was immunodetected as described above. The signal was compared to a standard curve obtained using a range of purified TcdA, from 0 to 0.5 μg (Supplementary Figs. S5a, S13) and normalized to the number of viable spores used. Taking into consideration that TcdA was only detected in about 54% of the 630Δ*erm* spores the amount of extractable, full-length TcdA per spore, on average, was estimated at a minimum of 9.9 × 10^2^ molecules (Supplementary Figs. S5 and S13). A minimum of about 7.3 × 10^2^ full-length TcdA molecules were extractable from the same number of R20291 spores (Supplementary Figs. S5, S13) but since TcdA was not detected by immunofluorescence in R20291 spores, the average number of molecules per spore could not be estimated.

We also extended our analysis to a group of characterized clinical strains of ribotypes 126 (strain E1), 053 (strain E7), 106 (strain E12), 014 (E14), 001/072 (strain E23), and 005 (strain E25)^81^. Spores produced by these strains were purified, fraccionated as above and the core/cortex and cortex/coat/exosporium proteins extracted, resolved by SDS-PAGE and the gels were subject to immunoblotting with the anti-TcdA antibodies. Spores of the non-toxinogenic E13 strain (RT017)^81^ were included as a specificity control for the antibody; as expected TcdA was mainly detected in the cortex/coat/exosporium fraction in spores of strain 630Δ*erm* whereas the toxin was not detected in any of the fraction prepared from E13 spores (Supplementary Figs. S6, S14), as expected from its genome sequence^81^. In contrast, TcdA was detected mainly in the core/cortex fraction of spores purified from strains E12, E14, E23, and E25 (Supplementary Figs. S6,S14). This analysis shows that the association of at least TcdA with spores is not specific to strains 630Δ*erm* and R20291, but also occurs in clinical isolates of several other ribotypes.

### The forespore or mother cell-specific expression of *tcdR* results in the association of TcdA with spores

That *tcdA* expression was detected both in the forespore and in the whole sporangium, suggested that expression during sporulation was required for the association of the toxin with spores. Because of protracted expression of *tcdA* in the mother cell and whole sporangia, however, to eliminate expression of the gene specifically in sporulating cells did not seem feasible. Therefore, we tested whether expression of *tcdA* in the forespore or the mother cell would be sufficient for the association of TcdA with spores, the *tcdR* deletion mutant was complemented in trans at the *pyrE* locus using a *tcdR* allele expressed from the forespore specific *sspA* promoter, or from the mother cell-specific *spoIIIAA* promoter^23–25^. We first examined expression of the P_*tcdA*_-*SNAP*^*Cd*^ fusion in the P_*sspA*_-*tcdR* (*tcdR*^FS^ for simplicity in Supplementary Figs. S7 and S15) and P_*spoIIIAA*_-*tcdR* (*tcdR*^MC^) complementation strains by fluorescence microscopy. In the *tcdR*^FS^ strain, expression of the fusion was detected in the forespore (Supplementary Fig. S7a, white arrowheads) whereas for the *tcdR*^MC^ strain a mother cell-only pattern of P_*tcdA*_-*SNAP*^*Cd*^ expression was detected (Supplementary Fig. S7a, blue arrowheads). Note that in the WT, a mother cell-only pattern of P_*tcdA*_-*SNAP*^*Cd*^ expression was not detected (see also above).

Biochemical fractionation of mature spores produced by the *tcdR*^MC^ strain shows that TcdA is present in the coat/exosporium fraction, at a level slightly higher than for the WT (Supplementary Figs. S7b and S15). TcdA is also present in the core/cortex fraction of *tcdR*^MC^ spores, but at levels lower than for the WT (Supplementary Figs. S7b and S15). In spores of the *tcdR*^FS^ strain, however, TcdA is present in the core/cortex fraction, but less extractable (or less abundant) than for WT or *tcdR*^MC^ spores, and absent or undetected in the coat/exosporium fraction (Supplementary Figs. S7b and S15). This suggests that forespore-produced TcdA is unable to reach the coat/exosporium (or it does so but below our detection level). It also suggests that most of the toxin detected in spores by immunofluorescence (Fig. 5a; see also above), results from the fraction of sporulating cells in which the σ^D^-dependent expression of *tcdA* persists in the mother cell. We further infer that the production of TcdA in the mother cell is sufficient for the association of the toxin not only with the coat/exosporium but also with the more internal core and/or cortex layers of spores.

### The spore-associated toxins remain functional

**Fig. 6:**
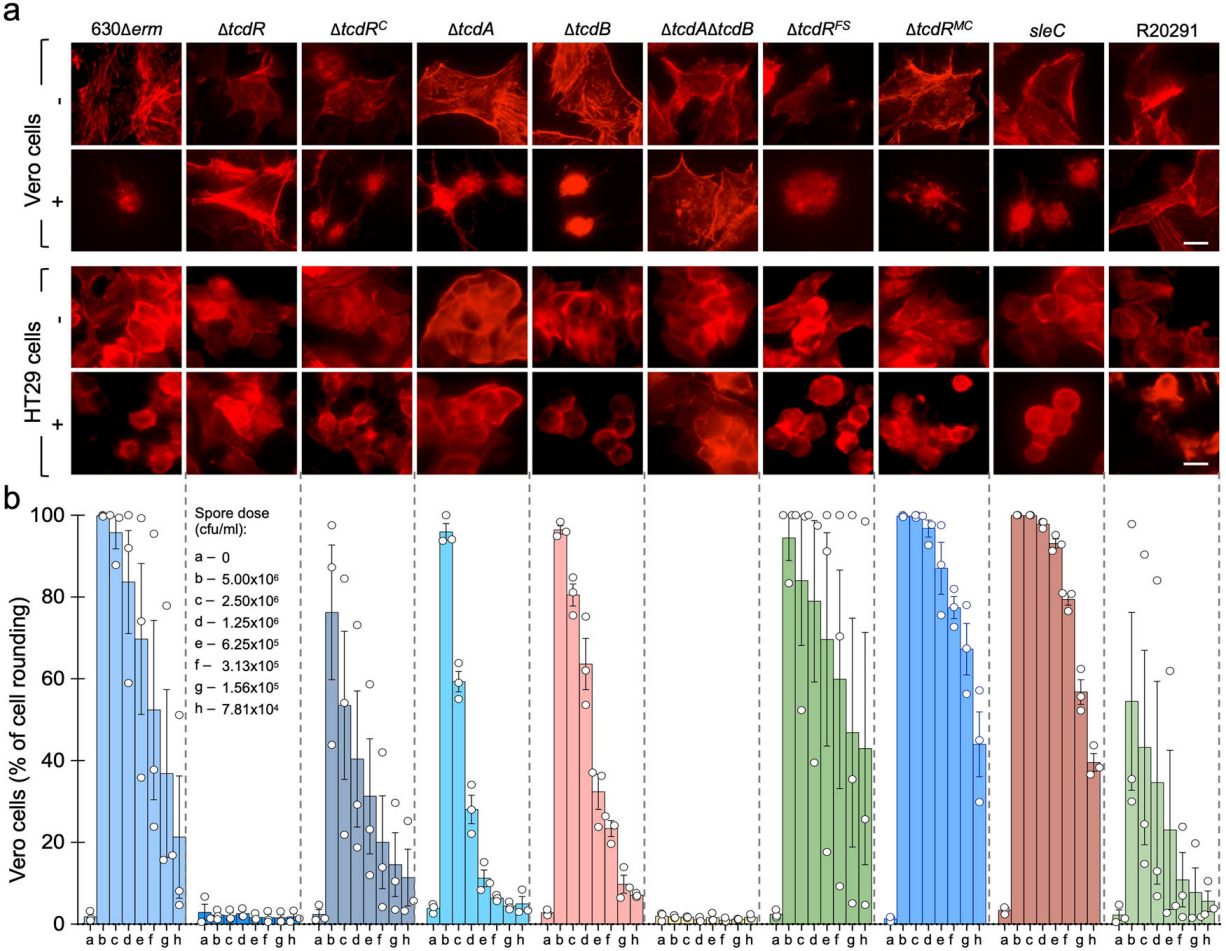
Spores produced by the 630Δ*erm* and R20291 strains have a cytopathic effect on epithelial cell lines. **a** Immunostaining of monolayers of Vero and HT29 cells. The cells were exposed to 5 × 10^6^ spores purified from sporulating cultures of the indicated strains. Following incubation for 24 h with spores, the cells were immunostained with Texas Red-Phalloidin to detect cell rounding. Scale bar, 10 μm. **b** Spores cause a dose-dependent cytopathic effect. Monolayers of Vero cells were exposed to the indicated number of spores (in CFU/ml) purified from each of the strains specified at the bottom. Following incubation, the cells were examined by phase contrast microscopy and the percentage of cell rounding scored for 220 cells (*n*). The experiment was performed in triplicate and error bars indicate the standard error.

The finding that at least TcdA associates with mature spores begged the question of its functionality. To test whether TcdA and TcdB, the latter of which we were not able to detect in spores by immunoblotting (above), remained functional, we conducted cytopathic assays using HT29 and Vero cell lines^82,83^. The toxins induce cell rounding, which can be monitored by microscopy^82,83^. HT29 and Vero cells, grown as monolayers, were exposed to different amounts of WT or Δ*tcdR* spores and cell rounding was monitored as a function of the spore concentration. We found that WT spores, but not those of the Δ*tcdR* mutant, caused cell rounding of both HT29 and Vero cells (Fig. 6a and Supplementary Fig. S8). This effect was dose-dependent for both cell lines (Supplementary Fig. S8) and shown in graphical form for Vero cells (Fig. 6b). Complementation with the wild-type *tcdR* allele at *pyrE* locus partially restored the cell-rounding ability to Δ*tcdR* spores (Fig. 6 and Supplementary Fig. S8). Thus, TcdA associates with spores in an active form. R20291 spores have a weaker cytopathic effect than 630Δ*erm* spores (Fig. 6 and Supplementary Fig. S8), possibly because of the more internal localization or lower abundance of TcdA, found mainly in the core/cortex fraction (Fig. 5c).

Both cell lines were exposed to spores produced by Δ*tcdA* and Δ*tcdB* single mutants (Fig. 6 and Supplementary Fig. S8). The Δ*tcdA* spores have a cytopathic effect in Vero cells, although lower than WT spores, since TcdB is present and the latter is known to cause cell rounding in this cell line^84^. However, these spores do not have a cytopathic effect in HT29 cells since this cell line is mainly susceptible to TcdA (Fig. 6)^84^. Δ*tcdB* spores have a cytopathic effect, again lower than WT spores, in both Vero and HT29 cells, consistent with the presence of TcdA in these spores and its reported cytopathic effect in both cell lines^84^.

Importantly, cell culture medium and cell culture supernatant did not induce spore germination, indicating that the toxin-induced cell rounding is caused by the spores. In line with this inference, *sleC* mutant spores were equally toxic against both HT29 and Vero cells when compared with WT 630Δ*erm* spores. Although they initiate germination, spores of a *sleC* mutant are not able to degrade the spore cortex and to complete the process^85^. Thus, germination does not need to be completed for the spore-associated cytopathic activity.

Spores produced by the *tcdR*^*FS*^ and *tcdR*^*MC*^ strains had a similar cytopathic effect against Vero cells and HT29 cells as those of the WT strain (Fig. 6). This suggests that when produced either in the forespore or the mother cell the toxins remain active when spore associated. That the cytopathic effect observed for *tcdR*^*FS*^ spores was the same as for *tcdR*^*MC*^ or WT spores is intriguing since in this strain TcdA shows a more internal, core and/or cortex localization. It seems likely, however, that differences in the structure of the spore surface layers between 630Δ*erm* and R20291 spores influence the exposure and activity of the spore-associated toxins.

## Discussion

The *tcdA* gene was found before to be expressed in the mother cell in a fraction of the sporulating cells^52,53^. We now show that other PaLoc genes are also expressed in sporulating cells according to different spatial patterns and regulatory schemes. We show that in a fraction of the sporulating cells, *tcdR*, *tcdA*, *tcdB* and *tcdE* are expressed only in the forespore, that *tcdA* and *tcdB* additionally show a whole-sporangium pattern of expression, *i.e*., both in the mother cell and in the forespore, and that the expression of *tcdC* only exhibits this latter pattern. We did not observe a mother cell-specific pattern of expression for any of the genes examined. In previous work, *tcdA* expression seemingly exhibited a mother cell-specific pattern but this could correspond to the whole sporangium pattern herein reported, because the fluorescence reporters used before, RFP, mSc and mNG, generally perform less well than the SNAP reporter in vegetative cells^59^ and possibly also in the forespore^52,53^. For this reason, it was also unclear from those studies whether forespore-specific expression of the PaLoc genes did occur.

We show that the whole sporangium pattern is both σ^D^- and TcdR-dependent, because the first triggers the auto-regulatory production of the second^52,53^. Expression of *tcdR*, and also of *tcdA*, which initially takes place in pre-divisional cells, persists in whole sporangia following asymmetric division, either because σ^D^ remains active and/or because sufficient TcdR is partitioned into the forespore and the mother cell. Heterogeneity in the population of sporulating cells with respect to whole sporangium *tcdA* expression most likely results from the TcdR auto-regulatory loop that operates in pre-divisonal cells^52^ (Fig. 2). In other words, the fraction of sporulating cells expressing the PaLoc genes reflects, at least in part, the fraction of ON vegetative cells and the bistable switch that controls their expression is propagated to whole sporangia. Thus, expression of the toxin-encoding genes in whole sporangia relies on the memory of a previous state, the vegetative state, referred to as hysteresis^86^.

We have shown that expression of *tcdR* is specifically induced in the forespore under the control of both σ^G^ and SpoVT and that *tcdA*, and presumably *tcdB* and *tcdE*, also shows TcdR-dependent, forespore-specific, expression. SpoVT enables σ^G^ to utilize a promoter that partially overlaps the σ^D^-dependent promoter. Expression of *tcdA*, *tcdB* and *tcdE* was only detected in a fraction of the forespores and TcdA was only detected in a fraction of spores. Possibly, the bistable switch controlling the expression of the PaLoc genes in vegetative cells is reproduced in the forespore with σ^G^ replacing σ^D^ in priming the auto-regulatory expression of *tcdR* and the expression of the PaLoc genes in a fraction of the forespores (Fig. 4f).

SpoVT is conserved among spore formers^20–22^ and positively regulates a subset of σ^G^-dependent genes in both *B. subtilis*, in *B. cereus* in *C. difficile*^25,87,88^. In *B. subtilis*, σ^G^ and SpoVT define coherent and incoherent feed-forward loops with AND gate logic that result in a pulse of expression of early σ^G^-dependent genes, and delayed expression of late genes, respectively^89^. σ^G^ and SpoVT may function in a similar way in *C. difficile* forming a coherent feed-forward loop with AND gate logic (Fig. 4f), that delays toxin production in the forespore until a late stage in development. SpoVT is a dimer of dimers, with each monomer formed by an N-terminal DNA binding domain and a C-terminal GAF (cGMP-specific and cGMP-stimulated phosphodiesterases, *Anabaena* adenylate cyclases, and *Escherichia coli* FhlA) domain. GAF domains act as sensory modules that can bind linear and cyclic nucleotides, porphyrin rings, as well as small signaling molecules such as homoserine lactones^90^. Whether SpoVT binds a specific ligand is unclear, as some GAF domains may function exclusively to mediate dimerization^90^. As yet unknown signals may control the activity of SpoVT and thus the level of toxin production in the forespore. Expression of *tcdR* and *tcdA* in the forespore additionally appears to be controlled through the activity of the late mother cell-specific regulator σ^K^. Yet, no cell-cell signaling pathway is known that links the activity of σ^K^ to that of σ^G^ in the forespore. One possibility is that the activity of σ^K^ is necessary to convey to the forespore signals that influence the activity of SpoVT.

**Fig. 7:**
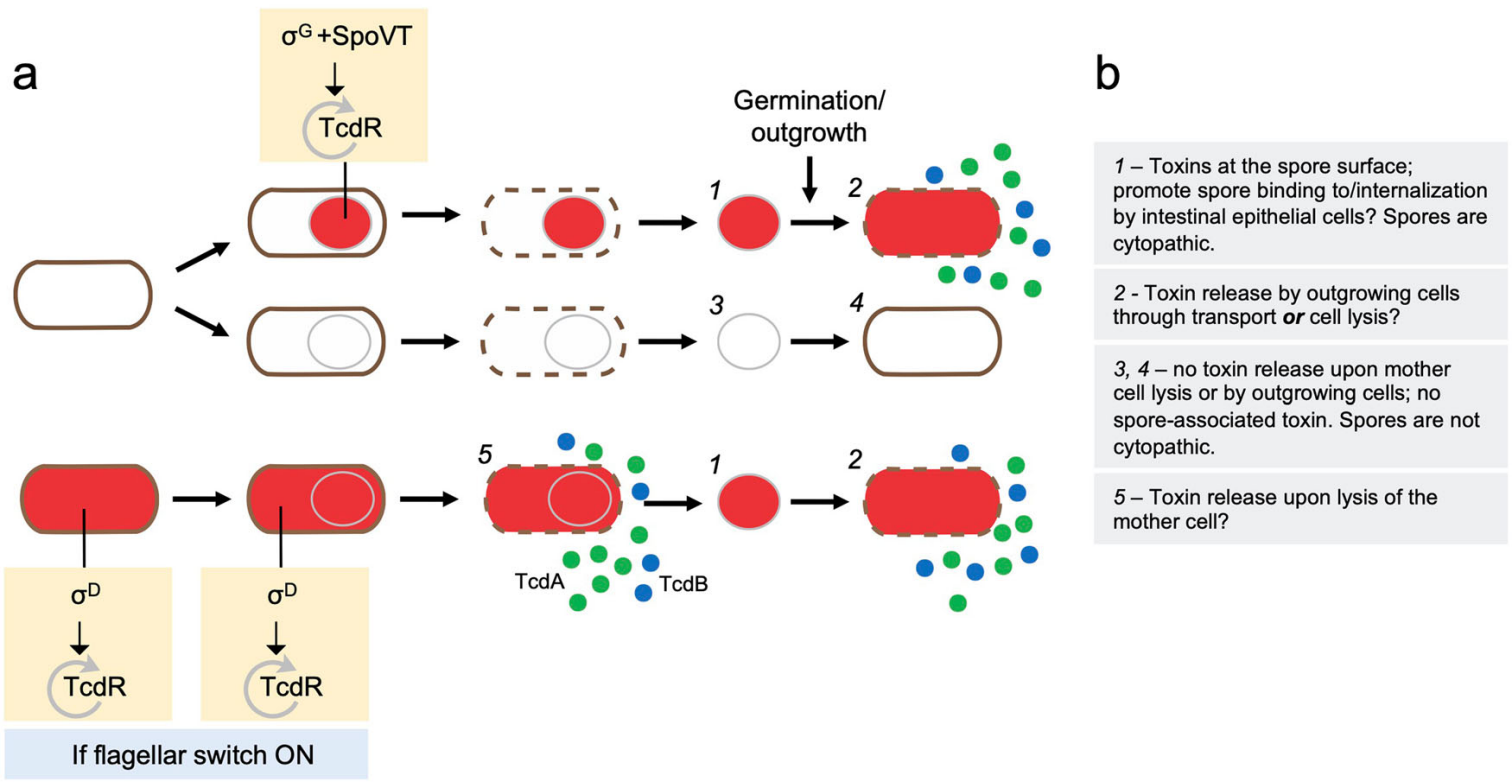
Functionally differentiated cell and spore populations. **a** Schematic representation of vegetative cells expressing the toxin-encoding genes (red filling) or not and their patterns of expression during sporulation (whole sporangium or forespore-specific). Five different possible functional classes (*1* to *5*) are highlighted with respect to the expression pattern of the toxin-encoding genes, the association of the toxins with spores and their release through lysis of the mother or following spore germination and outgrowth. The main regulatory proteins involved in the control of TcdR production are shown. If the flagellar switch is in the ON state^93^ σ^D^ primes the TcdR auto-regulatory loop in vegetative cells and in the mother cell during sporulation. In the forespore, however, the TcdR auto-regulatory loop is primed by σ^G^ together with SpoVT. **b** The expected properties of classes *1* to *5* are summarized in the panel.

We show that TcdA associates with spores and that expression of *tcdA* in sporulating cells, from either a forespore or a mother cell-specific promoter, is sufficient for this association (Supplementary Fig. S7). A study by Hong and co-authors showed that antibodies raised against a fragment of TcdA (residues 26-39 within the CROPs region) recognized species of about 100, 60, 50, and 20 kDa at the surface of *C. difficile* spores, and two of these species were identified as the aldehyde-alcohol dehydrogenase AdhE1 and the exosporium protein CdeC^91^. This study raised the possibility that coat/exosporium proteins cross-reacted with anti-TcdA antibodies. In our study, however, in which we used a monoclonal antibody that recognizes the TcdA CROPS region^92^, we detected a species of about 250 kDa, close to the expected size for TcdA, in WT but not in Δ*tcdA/*Δ*tcdB* mutant spores. Moreover, spores of both 630Δ*erm* and R20291 have a cytopathic effect when assayed against Vero or HT29 cells. Thus, TcdA associates in an active form with mature spores. In particular, fractionation and immunofluorescence studies showed the association of TcdA with the coat/exosporium and core/cortex of 630Δ*erm* spores, whereas in spores of the epidemic R20291 strain, TcdA is mainly associated with the spore core/cortex fraction. Importantly, TcdA was also found mainly in the core/cortex fraction of spore produced by clinical isolates of ribotypes 106, 014, 001/072, and 005 (Supplementary Fig. S6). This indicates that the association of the toxins with spores may be a general phenomenon. TcdA production in the mother cell results from protracted expression of σ^D^, which primes the TcdR auto-regulatory loop; σ^D^ production, in turn, is subject to a phase variation mechanism that controls the production of flagella and toxins^93^. The flagella switch involves inverted repeats that are conserved in all *C. difficile* strains that have been sequenced and carry the flagellar genes, but maybe locked in the ON state in strain 630Δ*erm*, in which the inverted repeats are shorter^93^. Since assembly of both the cortex and coat layers is mainly a function of the mother cell it is conceivable that some TcdA can associate with these layers during their formation but only in strains, such as 630Δ*erm* in which production of σ^D^ and TcdR is maintained in the mother cell. In the forespore, however, the TcdR auto-regulatory loops are primed by σ^G^ and SpoVT (Fig. 7). This may explain why in R20291 and the additional epidemic strains analyzed herein, TcdA is not detected in the cortex/coat/exosporium fraction while detected in the core/cortex fraction of all strains (Fig. 7).

Since that maintains expression of in the mother cell. Perhaps suggestively, TcdA has an affinity for glycan motifs with the core structure Gal β1-4 GlcNAc^8,28,94^ and it seems possible that TcdA associates with the cortex peptidoglycan via its CROPs region. Although we were unable to directly detect TcdB at the spore surface, the cytopathic effect of Δ*tcdA* spores on Vero cells, together with the lack of effect of Δ*tcdA*/Δ*tcdB* and Δ*tcdR* spores, suggests that TcdB also associates in active form with spores. Our finding that spores have a cytopathic effect on both Vero and HT29 cells is in line with the observation that spores induce the production of inflammatory cytokines and cause a cytotoxic effect on macrophages^95^.

The different patterns of *tcdR* and *tcdA* expression in sporulating cells indicate the existence of functionally different populations of cells and spores (Fig. 7). For spores that are formed in the host, it seems likely that mother cell expression provides an important route for toxin release upon lysis of this cell, coupling toxin release to the completion of spore development. The forespore-specific expression, of *tcdR* and *tcdA*, in turn, will likely result in the accumulation of the toxins in the forespore cytoplasm and presumably also in the cytoplasm of the outgrowing cell. Thus, for the fraction of forespores that produce them, the toxins could be quickly released upon spore outgrowth and since, as we show, *tcdE* is also expressed in the forespore, we speculate that *tcdE-*mediated release is involved (Fig. 7). It has been proposed that low levels of toxin cause inflammation, which in turn promotes colonization by vegetative cells during infection^96^. Thus, toxin release from mature spores or during spore germination and outgrowth might play a role in colonization. Cytopathic assays with a *sleC* null mutant suggest that germination does not need to be completed for spores to have a cytopathic effect (Fig. 6 and Supplementary Fig. S8). Still, we cannot rule out the possibility that the initial stages of germination^85^, with the consequent modifications of the spore surface, lead to some toxin release. In mice, the TcdA lethal dose is in the order of 50 ng and in challenge experiments using animal model around 100 spores are used^97–99^. It is difficult to correlate the amount of spore-associated toxin with the TcdA lethal dose and the amount of spore-delivered toxin will depend on the number of spores ingested. Low quantities of toxin, however, may still affect host cells; even sub-lethal concentrations of TcdA were shown to affect cell polarity, which in turn appears to facilitate access of the toxin to the host cell receptors, and therefore, colonization (^96^, reviewed in refs. ^8,28^).

The interaction of spores with E-cadherin promotes attachment to the colonic mucosa, and spore internalization, but whether additional spore receptors exist is unknown^13^. In contrast, several TcdA and TcdB receptors have been identified (^29^, reviewed by ref. ^8^). We speculate that spore-associated toxins may interact with their cognate receptors, thus contributing to spore binding to intestinal epithelial cells. TcdA and TcdB cause the redistribution of E-cadherin and increase spore binding to adherent junctions^57^. Thus, spore-associated TcdA and TcdB may promote spore binding to intestinal epithelial cells, in which case spore binding would be, to some extent, auto-regulatory. Presumably spore binding would also be independent of toxin production by a population of vegetative cells resulting from spore germination, which could be important for the initial interaction of infectious spores with the intestinal epithelial cells.

Importantly, in spores of the epidemic strain R20291, TcdA may have a more internal localization as compared to 630Δ*erm* spores (Fig. 5) perhaps explaining their lower cytopathic effect (Fig. 6 and Supplementary Fig. S8).

Toxins are known to be produced by sporulating cells in other pathogenic spore-formers: production of enterotoxin by *C. perfringens*, for example, is under the control of σ^E^ and σ^K^, which leads to accumulation of the toxin in the mother cell and its release into the intestinal lumen when the sporulating cells lyse^100–103^; also, one study reports on the association of a heat-resistant form of the botulin toxin with spores of *C. botulinum*^104^ and in several spore-forming entomopathogenic species such as *B. thuringiensis*, toxins are produced during sporulation and the insecticidal proteins associate with the exosporium as parasporal crystals (^105^ and references therein); in addition, and even though the toxin production factor AtxA negatively controls sporulation^106^, the protective antigen component of the anthrax toxin also associates with the exosporium and coat layers of *B. anthracis* spores^107^. These observations suggest that spore-forming pathogens, the expression of toxin genes by sporulating cells and the association of toxins with spores may represent a widespread strategy for both toxin release through lysis of the mother cell and for early delivery of the toxin.

## Methods

### Growth conditions and general methods

The *Escherichia coli* strain DH5α (Bethesda Research Laboratories) was used for molecular cloning, while HB101 (RP4) was used as the donor strain in *C. difficile* conjugation experiments^108^. Luria-Bertani medium was routinely used for growth and maintenance of *E. coli*. When appropriate, ampicillin (100 µg/mL) or chloramphenicol (15 µg/mL) was added to the culture medium. The *C. difficile* strains used in this study are congenic derivatives of the wild-type strain 630Δ*erm*^108^ or R20291 (Anaerobe Reference Laboratory, Cardiff, Wales, United Kingdom) and were routinely maintained anaerobically (5% H_2_, 15% CO_2_, 80% N_2_) at 37 °C in brain heart infusion (BHI) medium (Difco)^109^. For toxin assays and for sporulation assays, tryptone yeast extract (30 g/L tryptone; 20 g/L yeast extract) and sporulation medium (90 g/L tryptone; 5 g/L peptone; 1 g/L (NH_4_)_2_SO_4_; 1.5 g/L Tris base, pH 7.4) were used supplemented with cefoxitin (25 µg/mL) or thiamphenicol (15 µg/mL) when necessary. A defined minimal media (CDMM)^110^ with 1% agar was used as uracil-free medium when performing genetic selections. The minimal medium was supplemented with 5-Fluoroorotic acid (2 mg/mL) and uracil (5 µg/mL) when appropriate.

### *SNAP*^*Cd*^ transcriptional fusions and strain construction

The construction of the plasmids required for producing transcriptional *SNAP*^*Cd*^ fusions to the PaLoc promoters, deletion and mutational analysis of the *tcdR* promoter, *tcdR*, *spoVT*, *tcdA*, *tcdB* single and a *tcdA*/*tcdB* double mutant, to place *tcdR* under the control of the *sspA* or *spoIIIAA* promoters and for the overproduction of TcdR, σ^D^, σ^G^ and SpoVT in *E. coli* is described in the Supplementary Methods. Primers used for cloning or genome analysis are listed in Table 1. All plasmids are listed in Supplementary Table S2; all strains and their relevant properties are listed in Table S3.

**Table 1:** Oligonucleotides used in this work

Primer	Sequence (5′ to 3′)^a^
PtcdR-SNAP-EcoRI-Fw	CCCTTAAGCGAATTCTAAAAGTAAACG
PtcdR-SNAP-XhoI-Rev	CATAAAATCCTCGAGTCTTATATTTATAATG
PtcdA-SNAP-EcoRI-Fw	CACAAAGATGAATTCTGGTCAGTTGGT
PtcdA-SNAP-XhoI-Rev	GTATTATTACTCGAGATAATAAATCCAC
PtcdB-SNAP-EcoRI-Fw	TATCAAAGTGAATTCGTTTTTGAGGAAG
PtcdB-SNAP-XhoI-Rev	CTATAATACTCGAGCATCTAAATGCTAAAAC
PtcdC-SNAP-EcoRI-Fw	ACTTCACCTGAATTCTGGTATATTC
PtcdC-SNAP-XhoI-Rev	CATAATACAATCCTCGAGTTATTAGAT
PtcdE-SNAP-EcoRI-Fw	CAATTGGAATTCGATGGATATATGATATG
PtcdE-SNAP-XhoI-Rev	CATTCATCATAGCTCGAGTTTTTATTG
PtcdR_A_-SNAP-EcoRI-Fw	CCCCCCGAATTCTACTTTATTTATTAGAAAAA
PsigDmut-Fw	GCATATTTTCATATAAAATTTAATTTATTTGCATCTTCTATAATTATAATG
PsigDmut-Rev	CATTATAATTATAGAAGATGCAAATAAATTAAATTTTATATGAAAATATGC
TcdR-RBSopt-Fw	GCGCGCCTCGAG*GGAGGA*ACTACTATGCAAAAGTCTTTTTATG
TcdR-comp-HindIII-Rev PsspA-BamHI-Fw	CCCAAGCTTATTAATTTGCTCTTC AGATGAGGAGGATCCGGATAAAAGAGTTC
PsspA-SNAP-XhoI-Rev	CTTCCTTCTCTCGAGTTTATTTTGTGTTTGC
PspoIIIAA-BamHI-Fw	TAGATGGTGGGATCCCTAGGGCTTACCAAAAAAC
PspoIIIAA-SNAP-XhoI-Rev	GTTTATTCATCTCTTGCTCGAGTCCTTG
TcdR-comp-XhoI-Rev	CGCGCTCGAGATTAATTTGCTCTTC
TcdR-NcoI-Fw	CCCCCATGGAAAAGTCTTTTTATG
TcdR-SalI-Rev	CCCGTCGACCAAGTTAAAATAATTTTC
CDSigGpET28a-Fw	TGCCTCGAGTACATATTTTCTCATATTTTTTAAAGC
CDSigGpET28a-Rev	AGGGGGTGACCCCATGGCAGCTCTTAAATC
SigD-NcoI-Fw	CCCCCATGGATAGAGAAGAATTAATAAAAG
SigD-XhoI-Rev	CCCCTCGAGTATAGAATATTTAAGTTC
SpoVT-NdeI-Fw	CCCCATATGAAAGCAACAGGTATAGTTAG
SpoVT-XhoI-Rev	CCCCTCGAGTTATTGAACTTGTTTTCC
tcdR-AscI-Fw	CCCCGGCGCGCCATTATCTTAAGAGAGGAG
tcdR-SOE-Rev	CATAAATAAAATTTCTTGCAAATCATC
tcdR-SOE-Fw	TTGCAAGAAATTTTATTTATGGAAAATTATTTTAACTTG
tcdR-SbfI-Rev	CCCCCCTGCAGGTATCTATATAAATATCTG
tcdR-vef-Fw	GTATCATTTCACGAAGAGG
tcdR-vef-Rev	GGGTCATTTAAGTTTTCTC
tcdR-comp-BamHI-Fw	CCCGGATCCTAAAAATATTTTGATATG
tcdR-comp-HindIII-Rev	CCCAAGCTTATTAATTTGCTCTTC
tcdA-AscI-Fw	CCCCGGCGCGCCGGTAGTATATCAAACATTGG
tcdA-SOE-Rev	CTCATTTTCTCTTGGTCTAATGCTATATGCGAG
tcdA-SOE-Fw	CCAAGAGAAAATGAGCCTGGGATATATGGC
tcdA-SbfI-Rev	CCCCCCTGCAGGGATAAGGTTGTACTATGTAG
tcdB-AscI-Fw	CCCCGGCGCGCCCAAAGTAAGTCTGTTTTTGAGG
tcdB-SOE-Rev	CAATATTGCAACATATTCATCTTCTTG
tcdB-SOE-Fw	GAATATGTTGCAATATTGCAATTAGTG
tcdB-SbfI-Rev	CCCCCCTGCAGGGTCTTAAAAAATTGATAC
tcdA-vef-Fw	GATGGTGCATGGTCAGTTGG
tcdA-vef-Rev	GAAGATGGTGATGAGGTGC
tcdB-vef-Fw	GACAAGCTGTTAATAAGGC
tcdB-vef-Rev	CTGGTAATCCACATAAGCAC
YN3-vef-Fw	CATCAAGAAGAGCGACTTCG
YN3-vef-Rev	TTCTTTCTATTCAGCACTGTTATGC
pyrE-vef-Fw	CAATAATTTTATAACATTAACATGG
pyrE-vef-Rev	GTGTTACTTAAAAAATGTAAAT
YN4-vef-Fw	CAAGAAGAGCGACTTCGCGGAGCTGG
YN4-vef-Rev	CCATTACAGACTTATCCAGGG
SpoVT_sgRNA_Fw	TTTTCGTCGACAAGAATAGATGATCTTGGAAGTTTTAGAGCTAGAAATAGCAAGTTAAAATAAGGCTAGTCCGTTATCAACTTGAAAAAGTGGCACCGAGTCGGTGC
sgRNA_Rev	GCACCGACTCGGTGCCACTTTTTCAAGTTGATAACGGACTAGCCTTATTTTAACTTGCTATTTCTAGCTCTAAAACGTCGACGAAAA
spoVT-AscI-Fw	CCCCGGCGCGCCGAGAAAGATTTAGCAATG
spoVT-SOE-Rev	CTTTGAAACAACTACAATTGAACCCTCTTTTGGGATAACTACCCTTCC
spoVT-SOE-Fw	GGAAGGGTAGTTATCCCAAAAGAGGGTTCAATTGTAGTTGTTTCAAAG
spoVT-AsiSI-Rev	GATGGCGATCGCCTGCAACTTGAGACACAG
spoVT-vef-Fw	CGCGGATCCGATGAGTTTTTAAGAGAC
spoVT-vef-Rev	CGCGCTCGAGCAAAAGTCTGACCTAGAC
PsspA-EcoRI-FW	AGATGAGGAGAATTCGGATAAAAGAGTTCA
PsspA-SNAP-SOE-Rev	CATTTCACAATCTTTATCCATGTTGATTACCTTCCTTC
SNAP-SOE-Fw	ATGGATAAAGATTGTGAAATGAAGAGAACC
SNAP_C_-XhoI-Rev	CCCCTCGAGTTACCCAAGTCCTGGTTTCCCCAAACG

^a^Engineered restriction sites are underlined.

### SNAP induction assays in *E. coli*

In order to test for the ability of TcdR, σ^D^, σ^G^, or SigG together with SpoVT to induce transcription from the *tcdR* promoter, *E. coli* BL21 (DE3) strains bearing the plasmids for the overexpression of each of the proteins were co-transformed with either pMS464 or pCC27 (see Tables S2 and S3 and the Supplementary Information). The following strains were obtained that carry: pCC17 and pMS464 (AHED317) or pCC27 (AHEC319); pCC30 and pMS464 (AHEC320) or pCC27 (AHEC322); pFT36 and pMS464 (AHEC290), pCC27 (AHEC291) or pCC32 (AHEC853); pCC29 and pMS464 (AHEC314), pCC27 (AHEC316) or pCC32 (AHEC859). All strains were subjected to an auto-induction regime for the over-production of each protein or protein combinations^111^. The cultures were collected by centrifugation (4000 × *g*, for 10 min at 4 °C) and the cell sediment was suspended in PBS with 1 mM DTT. The cells were lysed using a French pressure cell (18,000 lb/in.^2^) and the extracts were incubated with 250 nM TMR‐Star substrate (New England Biolabs), for 30 min, in the dark. Proteins in the extracts were resolved by SDS-PAGE and labeled proteins were detected by fluoroimaging.

### SNAP labeling in *C. difficile* extracts

Samples (10 mL) were withdrawn from *C. difficile* TY cultures and the cells labeled with the TMR‐Star substrate (New England Biolabs), at a final concentration of 250 nM, for 30 min, in the dark. Following labeling, the cells were collected by centrifugation (4000 × *g*, for 5 min at 4 °C), the cell sediment was washed with phosphate-buffered saline (PBS) and suspended in 1 mL French press buffer (10 mM Tris pH 8.0, 10 mM MgCl_2_, 0.5 mM EDTA, 0.2 mM NaCl, 10% Glycerol, 1 mM PMSF). The cells were lysed using a French pressure cell (at 18,000 lb/in.^2^). Proteins in the extracts were resolved by SDS-PAGE (15% gels). The gels were first scanned in a TLA-510 fluorimager (Fuji), and then subject to immunoblotting with an anti-SNAP antibody (New England Biolabs) at a 1:1000 dilution; a rabbit secondary antibody conjugated to horseradish peroxidase (Sigma) was used at a dilution 1:10,000. The immunoblots were developed with enhanced chemiluminescence reagents (Amersham Pharmacia Biotech).

### Fluorescence microscopy and image analysis

For SNAP^Cd^ labeling, the TMR-Star substrate was added to cells in culture samples to a final concentration of 250 nM (New England Biolabs) and the mixture incubated for 30 min in the dark. Following labeling, the cells were collected by centrifugation (4000 × *g* for 5 min), washed four times with 1 mL of PBS, and finally suspended in 0.5 mL of PBS. For phase contrast and fluorescence microscopy, cells were mounted on 1.7% agarose-coated glass slides and observed on a Leica DM6000B microscope equipped with a phase contrast Uplan F1 100× objective and captured with a CCD Andor Ixon camera (Andor Technologies). Images were acquired and analyzed using the Metamorph software suite (version 5.8; Universal Imaging), cropped and adjusted using Adobe Photoshop.

### Immunofluorescence microscopy

For immunofluorescence analysis, the spores were fixed with 3% paraformaldehyde (pH 7.4) for 20 min in poly-L-lysine-coated glass cover slides. The fixed spores were rinsed three times with PBS and blocked with 1% bovine serum albumin (BSA) for 30 min. The slides were incubated overnight at 4 ^o^C with a monoclonal anti-TcdA primary antibody (Santa Cruz Biotechnology) (at a dilution of 1:5000). The slides were then incubated for 2 h at room temperature with Alexa Fluor 594 goat anti‐mouse IgG secondary antibody (Life Technologies) (1:500) in PBS–1% BSA, washed three times with PBS and once with distilled water (adapted from ref. ^75^).

### Spore production, purification, and fractionation

For spore production 10 mL of BHI media was inoculated with an isolated colony of *C. difficile* and cultured overnight at 37 ^o^C under anaerobic conditions. The next day, 150 mL of fresh BHI media was inoculated with 1.5 mL of the overnight culture and the new culture was incubated for 7 days at 37 ^o^C under anaerobic conditions. Cells were collected by centrifugation at 4800 × *g*, suspended in cold water and stored for 24 to 48 h at 4 ^o^C. The sediment was suspended in PBS with 0.1% Tween-20, and the spores were purified with a 42% Renografin (Bayer) step gradient^1^. The sediment, containing the spores, was suspended in PBS with 0.1% Tween-20, washed twice with the same buffer and twice with cold water. The final spore suspension was stored at −20 ^o^C until use. For spore fractionation, the spore coat was removed by suspending an amount of purified spores corresponding to an OD_580 nm_ of 2.0 (about 10^8^ spores), in 50 μL of decoating buffer (10% glycerol, 4% SDS, 10% β-mercaptoethanol, 1 mM DTT, 250 mM Tris pH 6,8). The spores were then boiled for 5 min and collected by centrifugation. The supernatant, corresponds to the coat/exosporium fraction. The spore sediment was washed twice with PBS with 0.1% Tween-20, and incubated with 50 mM Tris-HCl pH 8,0 with 2 mg/mL lysozyme for 2 h at 37 ^o^C to digest the spore cortex peptidoglycan and to release proteins associated with the spore core and cortex (core/cortex fraction). Proteins in the coat/exosporium and core/cortex fractions were resolved by SDS-PAGE (15% gels) and subject to immunoblotting with anti-CotD^5^, anti-TcdA (Santa Cruz Biotechnology), anti-GPR^6^, and anti-GerS^7^ antibodies.

### Sporulation assays

Overnight cultures grown at 37^o^C in BHI were used to inoculate BHI (at a dilution of 1:200). Once the OD_600_ reached 0.4, 100 μL the cultures were plated in TY plates and incubated for 24 h. After this incubation, the cells were scarped from the plates and suspended in 1 mL of TY. The suspension was serially diluted in TY and plated before and after heat treatment (30 min at 70 ^o^C), to determine the total and heat resistant colony forming units. The samples were plated as 20 μL spots in triplicate onto TY plates supplemented with 0.1% taurocholate (Carl Roth), to promote efficient spore germination.

### Toxin quantification by immunoblotting

For the quantification of the amount of TcdA associated with 630Δ*erm* and R20291 spores, samples of spore suspensions, corresponding to 10^8^ spores were fractionated into a coat/exosporium and core/cortex fractions as described in the preceding section. Samples were boiled for 5 min in 50 μL of decoating buffer (10% glycerol, 4% SDS, 10% β-mercaptoethanol, 1 mM DTT, 250 mM Tris pH 6,8) and loaded in the same gel together with purified TcdA (0.5 μg; 0.25 μg; 0.1 μg and 005 μg). TcdA was purified by Ni^2+^-affinity chromatography from cultures of *B. megaterium* carrying pHis-TcdA (^8^; the *B. megaterium* strain was a gift from Lacy Borden). TcdA was kept in 1X Phosphate buffer, pH 7.4. The gels were analyzed by immunobloting with an anti-TcdA antibody (Santa Cruz Biotechnology, as above). Pixels were quantified using Fiji-Image J and a linear regression was performed to calculate the amount of toxin in both the coat/exosporium and the core/cortex fractions. The experiment was performed in triplicate.

### Cell culture and cytopathic assays

Vero and HT29 cells were cultured in Dulbecco’s modified Eagle’s medium (Gibco) with GlutaMAX^TM^ supplemented with, 4.5 g/L Glucose, 5 or 10% (v/v) of bovine fetal serum for Vero or HT29 cells, respectively, and 1% (v/v) Pen-Strep (Sigma), at 37 ^o^C in a 5% CO_2_ atmosphere. Cells were grown until confluence in 96-wells plates and incubated with twofold serially diluted spores in Dulbecco’s modified Eagle’s medium, starting by 5×10^6^ purified spores^24^. After 24 h of incubation at 37 ^o^C in a 5% CO_2_ atmosphere, the cytopathic effect was monitored by phase contrast microscopy using a Nikon HCS microscope equipped with a 10× objective and an AndorZyla 4.2 sCMOS 4.2Mpx camera. The percentage of cell rounding was quantified on the phase contrast images.

### Actin immunostaining

For actin staining, Vero and HT29 cells with or without added spores were fixed in PBS containing 4% (w/v) paraformaldehyde for 15 min. Texas Red-conjugated Phalloidin (Thermo Fisher Scientific) was then added to a dilution of 1:100 in PBS-0.1% saponin containing 10% (v/v) horse serum. After staining, the cells were consecutively washed with PBS-0.1% saponin, PBS and ddH_2_O. The coverslips were assembled using Aqua-poly/Mount (Polysciences) on microscopy glass slides and the cells were examined by fluorescence microscopy on a Leica DM6000B microscope equipped with a phase contrast Uplan F1 63× objective and captured with a CCD Andor Ixon camera (Andor Technologies).

### Statistical analysis

Distributions obtained from quantifications of the SNAP^Cd^ labeling were statistically analyzed using the non-parametric Kolmogorov-Smirnov test. For the quantification of the fluorescence signal in different types of cells, three independent experiments were conducted. Only the results statistically significant (***, *p* > 0.001; ****, *p* < 0.0001) in all three experiments were considered statistically relevant.

### Reporting summary

Further information on research design is available in the Nature Portfolio Reporting Summary linked to this article.

### Supplementary information

cassona et al supplemental information revised

Description of additional supplementary files

Supplementary Dataset

Reporting summary

## Data Availability

Uncropped and unedited blot/gels are shown as follows: for Fig. 4d, e, see Supplementary Fig. S10; for Fig. 5b, c, see Supplementary Figs. S11 and S12; for Supplementary Fig. S2, see Supplementary Fig. S10; for Supplementary Fig. S5a, b, see Supplementary Fig. S13; for Supplementary Fig. S6a, b, see Supplementary Fig. S14; for Supplementary Fig. S7, see Supplementary Fig. S15. The Excel file provided (Supplementary data) contains the data used to make the plots shown in Figs. 2b, 3c, 4c and 6b.
