# Supplementary material for: Spores of Clostridioides difficile are toxin delivery vehicles
Source: Commun Biol. 2024 Jul 10;7:839. doi: 10.1038/s42003-024-06521-x (PMC11237016; doi:10.1038/s42003-024-06521-x)
Supplement: Supplementary file 1 — cassona et al supplemental information revised [file 42003_2024_6521_MOESM1_ESM.pdf]

Supplementary Information

Spores of *Clostridioides difficile* are toxin delivery vehicles

Carolina P. Cassona[1], Sara Ramalhete[1], Khira Amara[1], Thomas Candela[2], Imad Kansau[2], Cécile Denève-Larrazet[2], Claire Janoir-Jouveshomme[2], Jaime Mota[3,4], Bruno Dupuy[5], Mónica Serrano[1], Adriano O. Henriques[1]*

[1]Instituto de Tecnologia Química e Biológica, NOVA University Lisbon, Oeiras, Portugal;

[2]Micalis Institute, Université Paris-Saclay, INRAE, AgroParisTech, Jouy-en-Josas, France;

[3]Associate Laboratory i4HB, Institute for Health and Bioeconomy, NOVA School of Science and Technology, NOVA University Lisbon, Caparica, Portugal;

[4]CIBIO, Applied Molecular Biosciences Unit, Department of Life Sciences, NOVA School of Science and Technology, NOVA University Lisbon, Caparica, Portugal;

[5]Institut Pasteur, Université Paris-Cité, UMR-CNRS 6047, Laboratoire Pathogenèse des Bactéries Anaérobies, F-75015 Paris, France.

***Corresponding author**: Instituto de Tecnologia Química e Biológica, Universidade Nova de Lisboa, Avenida da República, Apartado 127, 2781-901 Oeiras, Portugal; Telephone: +351-21-4469521; Fax: +351-21-4411277; *e-mail*: aoh@itqb.unl.pt

Running title: Toxic spores of *C. difficile*

Keywords: PaLoc; *Clostridium difficile*; *Clostridium difficile* infection; sporulation; TcdA; TcdB

Subject terms: biological sciences/microbiology/pathogens

Supplementary Materials

Supplementary methods - Additional methods.

Supplementary Figure S1 - Sporulation and $SNAP^{Cd}$ labelling.

Supplementary Figure S2 - Assessing the extent of $SNAP^{Cd}$ labeling.

Supplementary Figure S3 - Deletion analysis of the *tcdR* regulatory region.

Supplementary Figure S4 - Expression of *tcdR* in sporulation mutants.

Supplementary Figure S5 - Quantification of spore-associated TcdA.

Supplementary Figure S6 - TcdA associates with spores of various ribotypes.

Supplementary Figure S7 - Forespore or mother cell-specific expression of *tcdR.*

Supplementary Figure S8 - Mature spores are have a cytopathic effect.

Supplementary Figure S9 - Original immunoblots and fluoroimaging scans for Figure S2.

Supplementary Figure S10 - Original gels and fluoroimaging scans for Figure 4d and e.

Supplementary Figure S11 - Original gels and immunoblots for Figure 5b.

Supplementary Figure S12 - Original gels and immunoblots for Figure 5c.

Supplementary Figure S13 - Original immunoblots for Figure S5.

Supplementary Figure S14 - Original gels and immunoblots for Figure S6.

Supplementary Figure S15 - Original immunoblots for Figure S7.

Supplementary Table S1 Heat resistant spore counts.

Supplementary Table S2 Plasmids used in this study.

Supplementary Table S3 Bacterial strains used in this study

Supplementary references

Supplementary methods

Transcriptional fusions of PaLoc promoters to the *$SNAP^{Cd}$* reporter. For the construction of transcriptional *$SNAP^{Cd}$* fusions to the promoters of the PaLoc genes *tcdR*, *tcdA*, *tcdB*, *tcdE* and *tcdC*, plasmid pFT47 [1] was used. The promoter regions of these genes were PCR-amplified using genomic DNA from *C. difficile* 630Δ*erm* with the primer pairs PtcdR-SNAP-EcoRI-Fw/ PtcdR-SNAP-XhoI-Rev, PtcdA-SNAP-EcoRI-Fw/ PtcdA-SNAP-XhoI-Rev, PtcdB-SNAP-EcoRI-Fw/ PtcdB-SNAP-XhoI-Rev, PtcdC-SNAP-EcoRI-Fw/ PtcdC-SNAP-XhoI-Rev and PtcdE-SNAP-EcoRI-Fw/ PtcdE-SNAP-XhoI-Rev (Table 1), to produce, 815, 520, 530, 430 and 600bp products, respectively. The promoter fragments were cloned between the EcoRI/ XhoI sites of pFT47 to yield pMS464, pMS470, pMS471, pMS472 and pMS473 (see S4 Table). The plasmids were introduced into *E. coli* HB101 (RP4) yielding strains AHEC125, AHEC144, AHEC145, AHEC146 and AHEC147 and then transferred to *C. difficile* 630Δ*erm* by conjugation, producing strains AHCD608 and AHCD668 through AHCD671 (Table S2).

Plasmid pMS464 was also introduced into the *C. difficile* 630Δ*erm* sporulation mutants *sigF* (AHCD533), *sigE* (AHCD532), *sigG* (AHCD534), *sigK* (AHCD535) and *spo0A* (AHCD536), yielding strains AHCD647 through AHCD651; pMS464 was also conjugated into a Δ*spoVT* mutant (our strain AHCD659; this work, see below) originating AHCD777. Additionally, pMS464 and pMS470 were conjugated into a Δ*sigD* mutant (AHCD687) producing strains AHCD780 and AHCD779, respectively.

Deletion and mutational analysis of the *tcdR* regulatory region. For deletion analysis of the *tcdR* promoter a fragment of 183 bp (fragment P_{tcdR-D}) was fused to *$SNAP^{Cd}$* in pFT47, according to the general strategy outlined above for the full-

length fusion (Fig. S3). The fragment was PCR-amplified using the primer pair P_{tcdR-A}-SNAP-EcoRI-Fw/ PtcdR-SNAP-XhoI-Rev and inserted between the EcoRI/XhoI sites of pFT47, yielding pCC20. The plasmid was introduced into *E. coli* HB101 (RP4) yielding strain AHEC237 and transferred to *C. difficile* 630Δ*erm* by conjugation, producing strain AHCD795.

To introduce point mutations at the -10 region of the σ^D promoter located upstream of *tcdR* (Fig. S3) overlap extension PCR was performed, initially using the primer pairs PtcdR-SNAP-EcoRI-Fw/ PsigDmut-Rev and PsigDmut-Fw/ PtcdR-SNAP-XhoI-Rev that originated fragments of 741 and 195 bp, and then, after mixing the two fragments, with PtcdR-SNAP-EcoRI-Fw/ PtcdR-SNAP-XhoI-Rev. The final PCR product was cloned between EcoRI/XhoI sites of pFT47 to give pCC27. The plasmid was transformed into *E. coli* HB101 (RP4) yielding strain AHEC274 and then transferred to *C. difficile* 630Δ*erm* by conjugation, producing strain AHCD821.

Construction of a Δ*tcdR* mutant through allele coupled exchange. Allele coupled exchange using a *pyrE* strain[2] was used to obtain in frame deletions of the *tcdR* gene in *C. difficile* 630Δ*erm* and R20291. *pyrE* negative strains previously constructed [2] were used in order to allow for a positive/negative selection. An allele exchange cassette was obtained by overlap extension PCR using the primer pair tcdR-AscI-Fw/tcdR-SOE-Rev for the left-hand homology arm (LHA) originating a 631 bp fragment and tcdR-SOE-Fw/tcdR-SbfI-Rev for the right-hand homology arm (RHA) originating a 522 bp fragment. The final PCR was performed with the primer pair tcdR-AscI-Fw/ tcdR-SbfI-Rev and the resulting fragments inserted between the SbfI and AscI sites of pMTL-YN3, to give pSR3 (for the construction of the Δ*tcdR* mutant) in 630Δ*erm*, and pMTL-YN4, to give pSR2 (for the construction of the Δ*tcdR* mutant in R20291). pSR3 and pSR2 were transformed into *E. coli* HB101

(RP4) to give AHEC239 and AHEC227, respectively, and then moved from these strains, by conjugation, into *C. difficile* 630Δ*erm*Δ*pyrE* and R20291Δ*pyrE*[2]. *C. difficile* transconjugants were selected by sub-culturing on BHI agar containing thiamphenicol (15 µg/mL) and cefoxitin (25 µg/mL) and re-streaked twice in this medium for 630Δ*erm* and four times for R20291. Colonies were screened by colony PCR with DNA obtained using 5% chelex resin (Sigma–Aldrich) to identify single-crossover mutants. For the *tcdR* gene the primer pairs YN3-vef-Fw/ tcdR-vef-Fw and YN3-vef-Rev/ tcdR-vef-Rev for 630Δ*erm* or YN4-vef-Fw/ tcdR-vef-Fw and YN4-vef-Rev/ tcdR-vef-Rev for R20291. The single-crossover mutants identified were streaked onto CDMM[3] with 1% agar supplemented with 5-Fluoroorotic acid (2 mg/mL) and uracil (5 µg/mL) in order to select for plasmid excision. The isolated FOA-resistant colonies were screened by colony PCR as above using the primer pair tcdR-vef-Fw/ tcdR-vef-Rev. The primers used flank the upstream and downstream homology regions. The double-crossover mutants that were thiamphenicol sensitive (AHCD811 in 630Δ*erm* and AHCD1093 in R20291) were reverted to a PyrE+ phenotype through conjugation with plasmid pMTL-YN1 for 630Δ*erm* and pMTL-YN2 for R20291. The resulting colonies were streaked onto non-supplemented CDMM agar and the restoration of the *pyrE* allele was confirmed by colony PCR using the primer pair pyrE-vef-Fw/pyrE-vef-Rev. Isolates with the expected structure were identified and named AHCD820 (Δ*tcdR* in 630Δ*erm*) and AHCD1102 (Δ*tcdR* in R20291).

Plasmids pMS470 and pMS464 (above; Table S3) were then introduced into the 630Δ*erm* Δ*tcdR* mutant (AHCD820) by conjugation with *E. coli* HB101 (RP4) derivatives, which resulted in strains AHCD840 and AHCD841 for Δ*tcdR*, respectively.

Construction of Δ*tcdA* and Δ*tcdB* mutants and of a Δ*tcdA*Δ*tcdB* double mutant through allele coupled exchange. Allelic exchange[2] was also used to construct Δ*tcdA* and Δ*tcdB* mutants and the double mutant Δ*tcdA*Δ*tcdB*. Plasmids pSR7 and pSR8 were used to delete *tcdA* and *tcdB*, respectively. For the construction of pSR7 the allele exchange cassettes were obtained by overlap extension PCR using the primer pairs tcdA-AscI-Fw/tcdA-SOE-Rev for the LHA originating a 626 bp fragment and tcdA-SOE-Fw/ tcdA-SbfI-Rev for the RHA originating a 544 bp fragment. The final PCR was obtained with the primer pair tcdA-AscI-Fw/ tcdA-SbfI-Rev. For the construction of pSR8, the allele exchange cassettes were obtained by overlap extension PCR using the primer pairs tcdB-AscI-Fw/ tcdB-SOE-Rev for the LHA originating a 635 bp fragment and tcdB-SOE-Fw/ tcdB-SbfI-Rev for the RHA originating a 497 bp fragment. The final PCR was obtained with the primer pair tcdB-AscI-Fw/ tcdB-SbfI-Rev. All plamids were verified by DNA sequencing. The plasmids obtained were transformed into *E. coli* HB101 (RP4) yielding strains AHEC498 (for Δ*tcdA*) and AHEC499 (for Δ*tcdB*). The *E. coli* strains for the construction of the Δ*tcdA* and Δ*tcdB* mutants were subsequently mated with *C. difficile* 630Δ*erm*Δ*pyrE* and transconjugants were selected by sub-culturing on BHI agar containing thiamphenicol (15 µg/mL) and cefoxitin (25 µg/mL) and re-streaked twice in this medium. Colonies were screened by colony PCR with DNA prepared using 5% chelex resin (Sigma-Aldrich) to identify single-crossover mutants. For the *tcdA* gene the primer pairs YN3-vef-Fw/ tcdA-vef-Fw and YN3-vef-Rev/ tcdA-vef-Rev were used, while for *tcdB* the primer pairs YN3-vef-Fw/ tcdB-vef-Fw and YN3-vef-Rev/ tcdB-vef-Rev were used. The single-crossover mutants identified were streaked onto CDMM[3] with 1% agar supplemented with 5-Fluoroorotic acid (2 mg/mL) and uracil (5 µg/mL) in order to select for plasmid

excision. The isolated FOA-resistant colonies were screened by colony PCR using the primer pair tcdA-vef-Fw/tcdA-vef-Rev for *tcdA* and tcdB-vef-Fw/tcdB-vef-Rev for *tcdB*, which flank the upstream and downstream homology regions.

The double-crossover mutants that were thiamphenicol sensitive (AHCD999 for *tcdA* and AHCD1415 for *tcdB*) were reverted to a PyrE+ phenotype through conjugation with the plasmid pMTL-YN1. The resulting colonies were streaked onto non-supplemented CDMM agar and the restoration of the *pyrE* allele was confirmed by colony PCR using the primer pair pyrE-vef-Fw/pyrE-vef-Rev. This produced strains AHCD1002 (Δ*tcdA*) and AHCD1439 (Δ*tcdB*). The double-crossover Δ*tcdA* mutant that was thiamphenicol sensitive (*pyrE-)* was used to mate with *E. coli* AHEC499 to construct the double Δ*tcdA*/Δ*tcdB* mutant AHCD1046, using the same methodology outlined above for the single mutant. This strain was reverted to a PyrE+ phenotype through conjugation with pMTL-YN1. The resulting colonies were streaked onto non-supplemented CDMM agar and the presence of a restored *pyrE* allele was confirmed by colony PCR using the primer pair pyrE-vef-Fw/ pyrE-vef-Rev. This screen led to the isolation of strain AHCD1066.

Complementation through allelic exchange at the *pyrE* locus. In order to complement the Δ*tcdR* mutation, the coding sequence of the *tcdR* gene and its promoter region, a total of 1460 bp, was PCR amplified from 630Δ*erm* or R20291 genomic DNA, using the primer pair tcdR-comp-BamHI-Fw/tcdR-comp-HindIII-Rev and the fragment inserted between the BamHI and HindIII sites of either pMTL-YN1C, yielding pSR5 (for 630Δ*erm*) and pMTL-YN2C, yielding pSR34 (for R20291). pSR5 and pSR34 were transformed into *E. coli* HB101 (RP4) yielding strains AHEC285 and AHEC661, respectively, and subsequently mated with *C. difficile* 630Δ*erm*Δ*pyrE*Δ*tcdR* and R20291Δ*pyrE*Δ*tcdR*. The transconjugants obtained were

streaked onto CDMM and tested by colony PCR using primer pair tcdR-vef-Fw/ tcdR-vef-Rev to test for both *tcdR* complementation and with primer pair pyrE-vef-Fw/pyrE-vef-Rev for *pyrE* reversion; clones with the expected changes were identified and named AHCD828, for 630Δ*erm*, and AHCD1161, for R20291.

Plasmids pMS470 and pMS464 (above; Table S3) were introduced into the complementation strain through matting from *E. coli* HB101 (RP4) derivatives, resulting in strains AHCD845 and AHCD846, respectively.

Placing *tcdR* under the control of forespore- or mother cell-specific promoters. The *sspA* or *spoIIIAA* promoters were fused to the *tcdR* gene using overlap extension PCR. The *tcdR* gene was PCR amplified from C*. difficile* 630Δ*erm* genomic DNA with an optimized RBS using the primer pair TcdR-RBSopt-Fw/ TcdR-comp-HindIII-Rev, producing a 723bp. The PCR fragment was inserted between the XhoI/HindIII sites of pMTL-YN1C, to produce pCC44. The *sspA* and *spoIIIAA* promoter regions were PCR amplified using primer pairs PsspA-BamHI-Fw/ PsspA-SNAP-XhoI-Rev and PspoIIIAA-BamHI-Fw/ PspoIIIAA-SNAP-XhoI-Rev, producing fragments of 482 and 494 bp which were inserted between the BamHI/XhoI sites of pCC44 to give pCC45 (P_{sspA}-*tcdR*) and pCC46 ($P_{spoIIIA}$-*tcdR*). The plasmids were transformed into *E. coli* HB101 (RP4) yielding strains AHEC391 and AHEC389, respectively, and subsequently transferred to *C. difficile* 630Δ*erm*Δ*pyrE*Δ*tcdR* by conjugation.

The transconjugants obtained were streaked onto CDMM and tested by colony PCR using the primer pair PsspA-BamHI-Fw/ TcdR-comp-XhoI-Rev and PspoIIIAA-BamHI-Fw/ TcdR-comp-XhoI-Rev to test for integration and with the primer pair pyrE-vef-Fw/pyrE-vef-Rev to test for *pyrE* reversion, yielding strains AHCD900 (P_{sspA}-*tcdR*) and AHCD926 ($P_{spoIIIA}$-*tcdR*), respectively.

Construction of a Δ*spoVT* in-frame deletion mutant using CRISPR-Cas9. A CRISPR-Cas9-based approach[4] was used for the in-frame deletion of the *spoVT* gene in the *C. difficile* 630Δ*erm sigG* mutant previously constructed using the ClosTron system[1]. The sgRNA, used to target the Cas9 nuclease to the *spoVT* gene, was obtained by a primer dimer PCR reaction. The primers for amplification were designed by adding the 20 nucleotides SEED region identified with the Benchling software (https://benchling.com/crispr) to the universal forward primer (SpoVT_sgRNA_Fw) together with the universal reverse primer sgRNA_Rev. The allele exchange cassette composed of a left-hand homology arm (LHA) and a right-hand homology arm (RHA) was obtained by overlap extension PCR using the primer pair spoVT-AscI-Fw/spoVT-SOE-Rev for the LHA originating a 701 bp fragment and spoVT-SOE-Fw/spoVT-AsiSI-Rev for the RHA originating a 709bp fragment. The final PCR was done with the primer pair spoVT-AscI-Fw/spoVT-AsiSI-Rev originating a 1410 bp fragment that was inserted between the AscI/AsiSI sites of pMTL-Cas9[4] together with the sgRNA fragment that was cloned between the SalI/AsiSI sites, to yield pCC86, the sequence of which was verified. pCC86 was introduced into *E. coli* HB101 (RP4) to give AHEC855 and then introduced into *C. difficile* 630Δ*erm sigG* by conjugation. Transconjugants were selected by sub-culturing on BHI agar containing thiamphenicol (15 µg/mL) and cefoxitin (25 µg/mL) and then re-streaked in this medium. The selected transconjugants were screened by colony PCR using the primer pair spoVT-vef-Fw/spoVT-vef-Rev. A strain with the expected structure was isolated and named AHCD1418.

Plasmids and strains for the overproduction of TcdR, σ^D, σ^G and SpoVT in *E. coli*. For TcdR overproduction, *tcdR* was amplified from C.*difficile* 630Δ*erm* genomic DNA using the primer pair TcdR-NcoI-Fw/ TcdR-SalI-Rev that produced a

551bp fragment and cloned between the NcoI/SalI sites in pFN127 (pET16b-Strep tagII; Amp^R), originating pCC17. For σ^G overexpression, *sigG* was amplified from C*.difficile* 630Δ*erm* genomic DNA using the primer pair CDSigGpET28a-Fw/ CDSigGpET28a-Rev that produced a 774bp fragment and cloned between the NcoI/XhoI sites in pET28a, originating pFT36. For SpoVT overexpression, *spoVT* was amplified using the primer pair SpoVT-NdeI-Fw/ SpoVT-XhoI-Rev producing a 540bp fragment and cloned between the NdeI/XhoI sites in pET16b originating pCC22. For σ^G and SpoVT co-induction, *sigG* was cut from pFT36 using NcoI/XhoI and cloned NcoI/SalI in pETDuet and *spoVT* was cut from pCC22 using NdeI/XhoI and cloned using the same restriction sites in pETDuet already containing *sigG*, producing pCC29. For σ^D overproduction, *sigD* was amplified from using the primer pair SigD-NcoI-Fw /SigD-XhoI-Rev and cloned between the NcoI/XhoI sites in pET28a, originating pCC30. All plasmids were transformed into *E. coli* BL21 (DE3) to yield strains AHEC217, AHEC068, AHEC304 and AHEC313. Additionally, pMS464 and pCC27 were co-transformed into *E. coli* BL21 (DE3) together with pFT36, pCC29 or pCC17, yielding strains AHEC290, 291, 314, 316, 317 and 319 (see Tables S2 and S3). Furthermore, pMS464 was co-transformed with pCC30 to produce AHEC320. As a positive control a fusion was constructed between the *sspA* promoter and $SNAP^{Cd}$ using overlap extension PCR. First, primers PsspA-EcoRI-Fw/ PsspA-SNAP-SOE-Rev were used to amplify the *sspA* promoter region producing a 498bp fragment. Secondly, primer pair SNAP-SOE-Fw/ $SNAP_C$-XhoI-Rev was used to amplify $SNAP^{Cd}$ from pFT47 producing a 550bp fragment. Finally, primer pair PsspA-EcoRI-Fw / $SNAP_C$-XhoI-Rev produced a fragment with 1048bp, which was cloned between the EcoRI/ XhoI sites in pMTL84121, yielding pCC32.

Plasmid pCC32 was co-transformed into *E. coli* BL21 (DE3) together with pFT36 or pCC29, yielding strains AHEC853, and AHEC859, respectively.

Supplementary figures and legends

Supplementary Figure S1

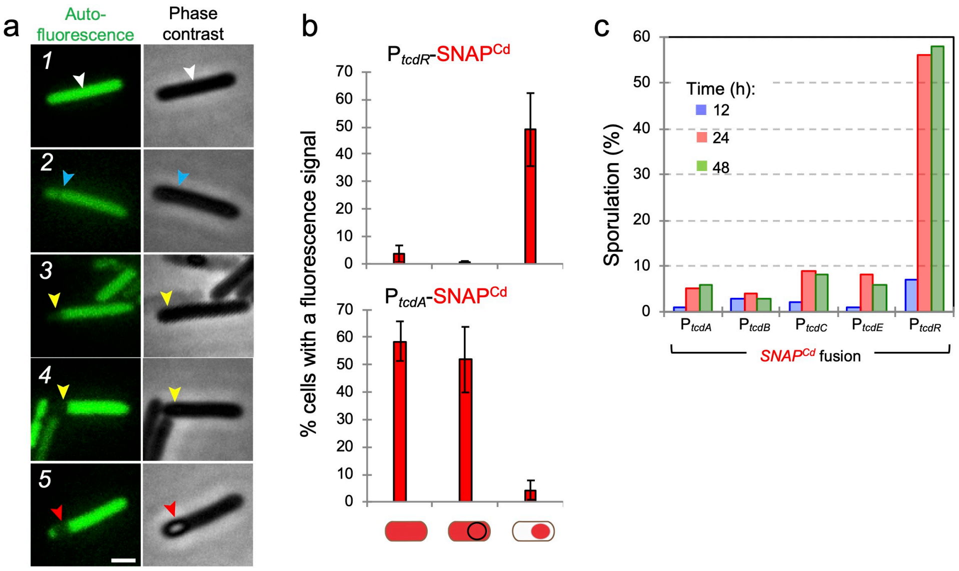

Fig. S1 - Sporulation and $SNAP^{Cd}$ labelling.a: Phase contrast and fluorescence microscopy of of vegetative (*1*) or sporulating cells (*2* to *5*) of strain 630Δ*erm* in BHI. The autofluorescence patterns together with phase contrast imaging allows the identification of vegetative cells and cells at different stages of sporulation: *1*, vegetative cells; *2*, a cell with an asymmetric division septum (the blue arrowhead points to the division septum); *3*, *4*, cells during engulfment (the yellow arrowhead indicates the position of the forespore); *5*, following engulfment completion (the red arrowhead points to the forespore). Scale bar, 1 μm. **b**: Shows the percentage of cells with the indicated patterns of P_{tcdR}- and P_{tcdA}-$SNAP^{Cd}$ expression scored 24 h after inoculation in BHI. The following patterns of expression were scored (as indicated at the x axis): in vegetative cells, the whole sporangium and forespore-specific. **c**: The panel shows the efficiency of sporulation (as the percentage of heat resistant spores versus total viable cells) for strains bearing the indicated *$SNAP^{Cd}$* fusions, measured 12, 24, and 48 hours after inoculation in TY medium.

Supplementary Figure S2

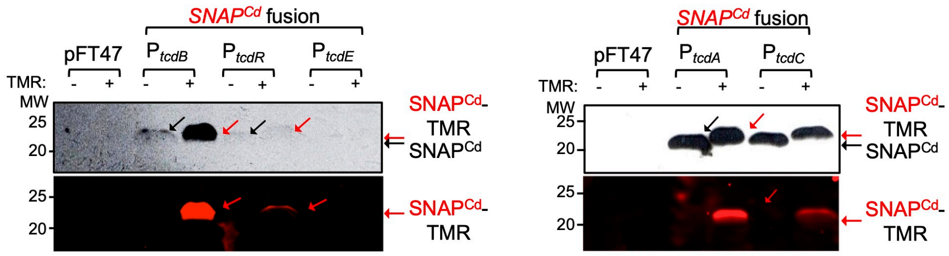

Fig. S2 - Assessing the extent of SNAP^{Cd} labeling. Whole cell extracts were prepared from derivatives of strain 630Δ*erm* bearing the indicated plasmids or fusions, before ("-" sign) or immediately after ("+") labeling with TMR-Star. The indicated amount of total protein was resolved by SDS-PAGE, and subject to immunoblotting with an anti-SNAP antibody (top). Duplicate gels were scanned on a fluoroimager (bottom). Black and red arrows point to unlabeled or TMR-Star-labeled SNAP^{Cd}, respectively. Note that TMR-Star forms a covalent complex with SNAP^{Cd}, which migrates slower than the unlabeled SNAP^{Cd}. Thus, complete labeling of the is indicated by a red band in the fluoroimage that corresponds to a single, slower migrating band in the immunoblot, *i.e.*, no band corresponding to the unlabeled SNAP^{Cd} is detected. Strain 630Δ*erm* carrying pFT47 (empty vector) was used as a negative control for SNAP^{Cd} production. The position of molecular weight markers (in kDa) is indicated on the left side of the panels.

Supplementary Figure S3

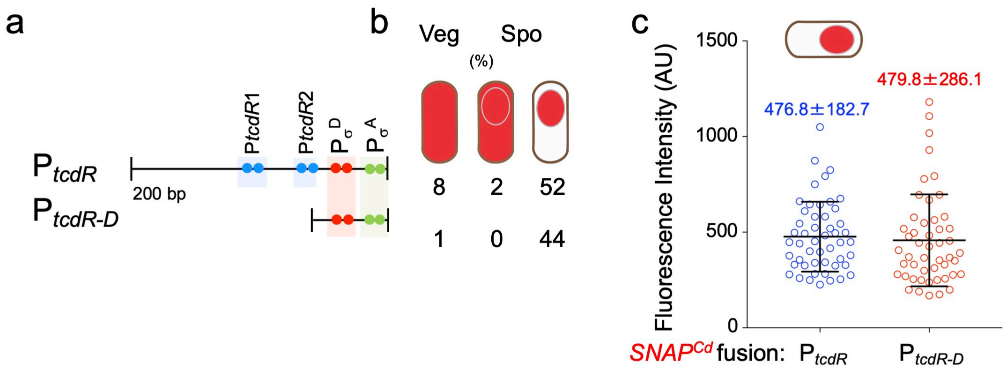

Fig. S3 - Deletion analysis of the *tcdR* regulatory region. a: Schematic representation of the fragments from the *tcdR* regulatory region, P_{tcdR} (see also Figure 1) or $P_{tcdR\text{-}D}$, fused to the reporter $SNAP^{Cd}$. P_{tcdR1} and P_{tcdR2} represent the promoters recognized by TcdR (blue dots), P_{σ}^{D} represents the promoter recognized by σ^{D} (red dots) and the green dots show the position of a σ^{A}-type promoter. Each construct was inserted in strain 630Δ*erm* (WT). **b:** Percentage of vegetative (Veg) or sporulating cells (Spo) with the indicated pattern of $SNAP^{Cd}$ signal. The number of cells analyzed for each fusion, n, is as follows: P_{tcdR}-$SNAP^{Cd}$, n = 371; $P_{tcdR\text{-}D}$-$SNAP^{Cd}$, n = 584. **c:** Quantitative analysis of the fluorescence intensity (Arbitrary Units, AU) of the $SNAP^{Cd}$ signal per forespore for strains expressing the P_{tcdR} and $P_{tcdR\text{-}A}$ fusions represented in **a**. The numbers in the panels represent the mean value ± the standard deviation.

Supplementary Figure S4

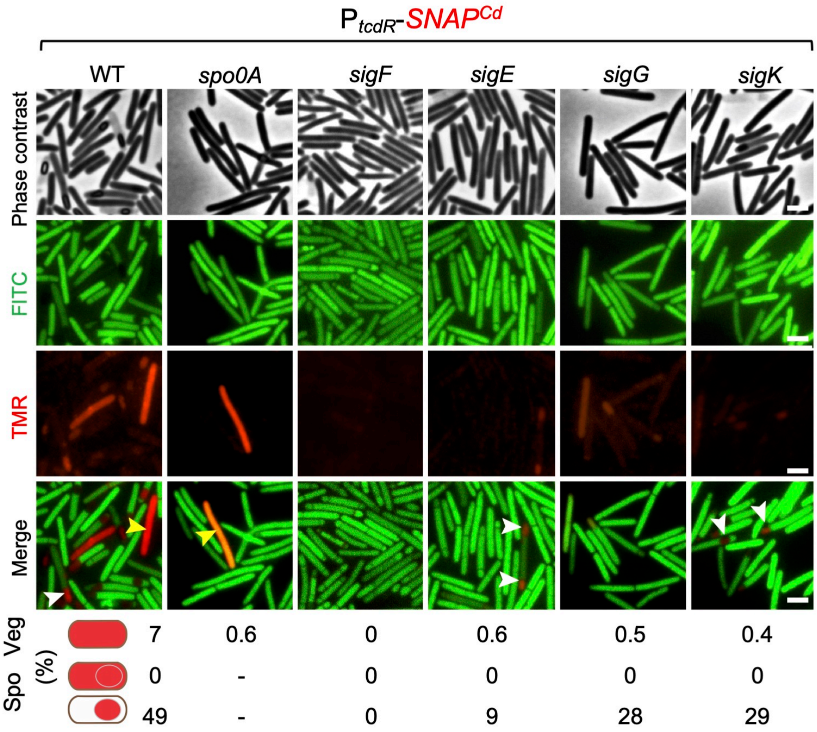

Fig. S4 - Expression of *tcdR* in sporulation mutants. Microscopy analysis of *C. difficile* cells carrying the fusion of the *tcdR* promoter to $SNAP^{Cd}$ in strain 630Δ*erm* (WT) and in the *spo0A*, *sigF*, *sigE*, *sigG* and *sigK* mutants. The cells were collected after 24h of growth in TY liquid medium, labeled with TMR-Star and examined by phase contrast and fluorescence microscopy to monitor $SNAP^{Cd}$ production. The merged images show the overlap between the TMR-Star (red) and the auto-fluorescence (green) channels. The images are representative of the expression patterns observed for the different fusions. The white arrowheads show sporangia with forespore-specific $SNAP^{Cd}$ expression (including the diasporic cell in the *sigE* mutant); yellow arrowheads show vegetative cells with expression. Scale bar, 1 µm. Expression patterns were scored as described in the legend for S3 figure and their percentages are indicated below the fluorescence images. Number of cells, n, scored for each strain: WT, n = 983; *spo0A*, n = 1137; *sigE*, n = 1256; *sigG*, n = 1323; *sigK* = 906.

Supplementary Figure S5

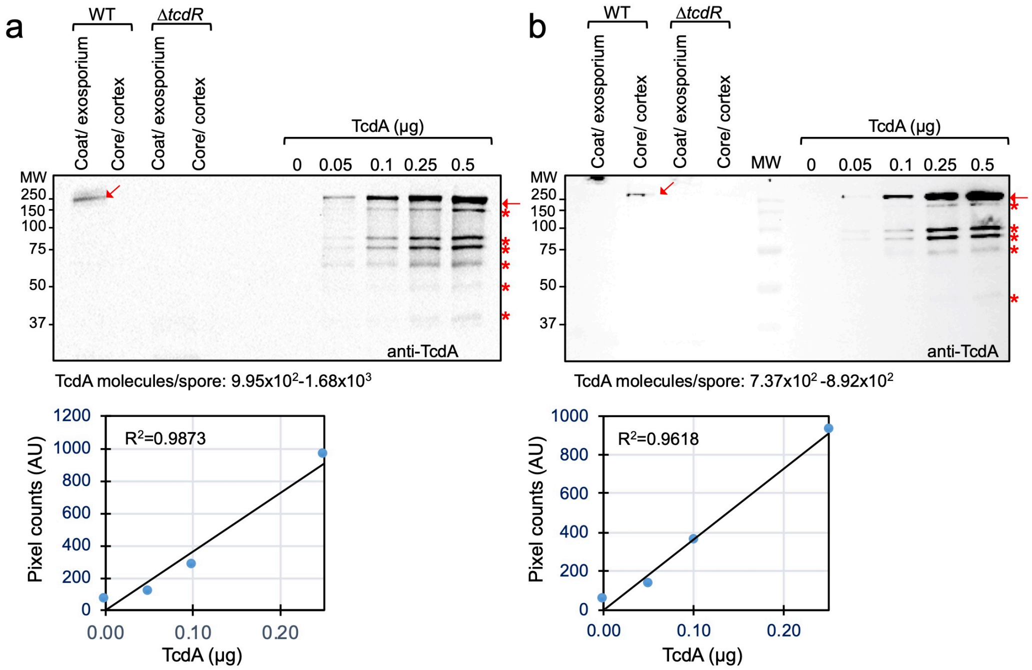

Fig. S5 - Quantification of spore-associated TcdA. Quantitative immunoblotting of the spore associated TcdA toxin. Purified spores produced by strains 630Δ*erm* and Δ*tcdR* (**a**) and R20291 and a congenic Δ*tcdR* mutant (**b**) were fractionated into a coat/exosporium and a core/cortex fractions. Extracted proteins were resolved by SDS-PAGE along with increasing concentrations of purified TcdA, as indicated. The gels were then subject to immunobloting with an anti-TcdA antibody. The red arrow points to the position of full-length TcdA; other bands (red asterisks) are likely degradation products of TcdA. The position of MW markers (in kDa) is shown on the left side of the panels. The graphs below each panel show a plot of the pixel counts obtained as a function of the amount of full-length TcdA, and the R^2 value is shown.

Supplementary Figure S6

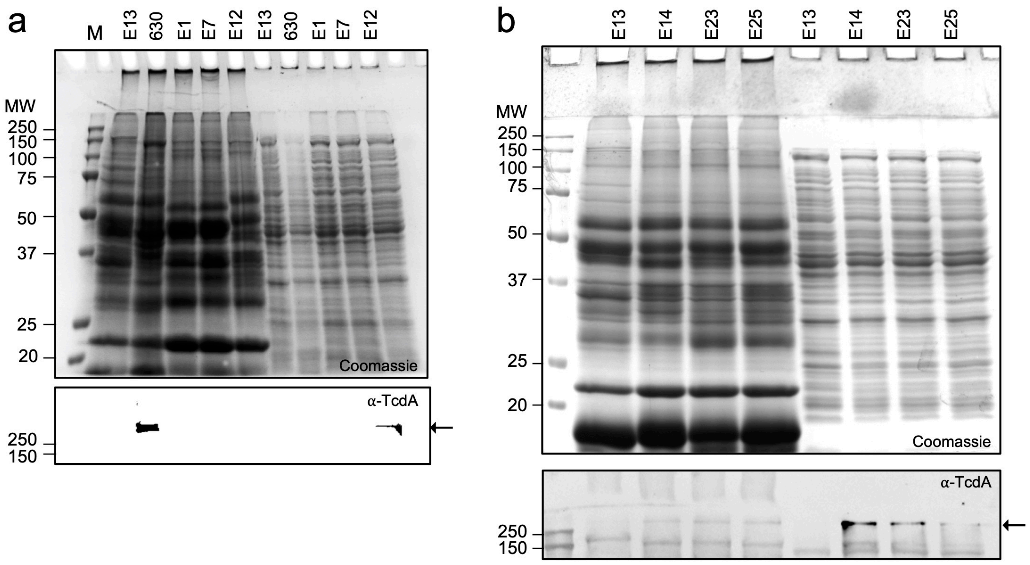

Fig. S6 - TcdA associates with spores of various ribotypes. **a**: Fractionation of spores produced by the indicated strains: **a**, E13, 630Δ*erm* (630), E1, E7, E12; **b,** E13, E14, E23, E25; Purified spores were fractionated into a cortex/coat/ exosporium and a core/cortex fraction. The proteins in the various fractions were resolved by SDS-PAGE (top) and subject to immunoblotting (bottom) with an anti-TcdA. Strain E13, a non-toxin producer was used as a control for the specificity of the anti-TcdA antibody. The arrows point to the position of the main form of TcdA. The position off molecular weight markers (in kDa) is shown on the left side of the panels.

Supplementary Figure S7

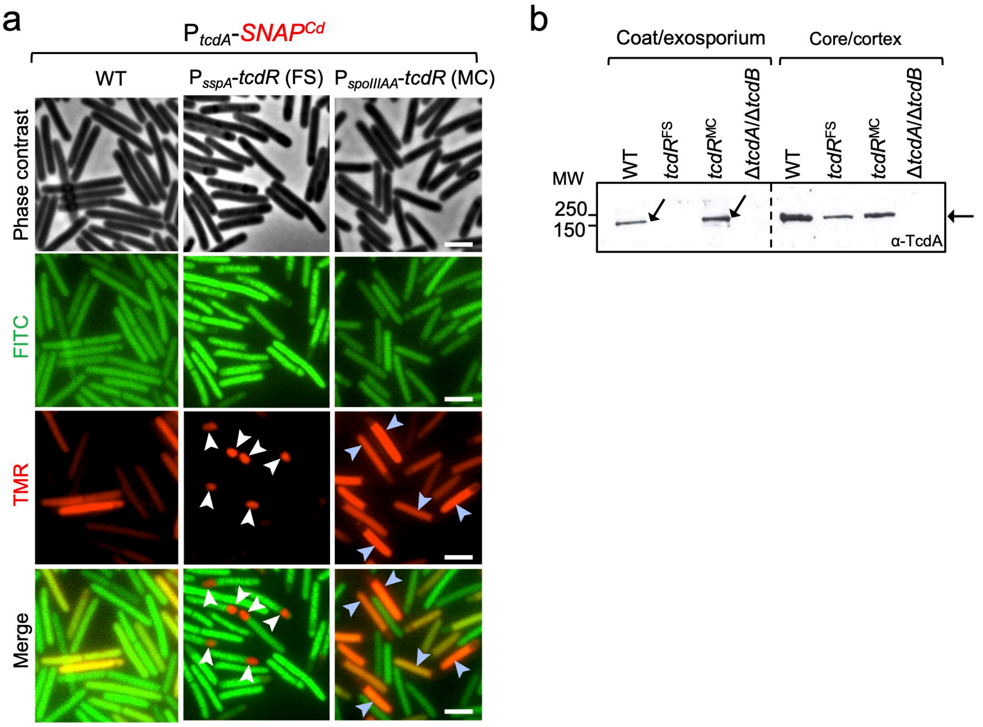

Fig. S7 - Forespore or mother cell-specific expression of *tcdR*. a: The pattern of P$_{tcdA}$-*SNAP*^{Cd} expression when *tcdR* is under the control of the forespore-specific *sspA* promoter (*tcdR*^{FS}) or the mother cell-specific *spoIIIAA* promoter (*tcdR*^{MC}). The cells were collected after 24h of growth in TY liquid medium, labeled with TMR-Star and examined by phase contrast and fluorescence microscopy to monitor SNAP^{Cd} production. The merged images show the overlap between the TMR-Star (red) and the auto-fluorescence (green) channels. In the P$_{sspA}$-*tcdR* (FS) column, the white arrowheads point to sporangia with forespore-specific expression of SNAP^{Cd}; in the P$_{spoIIIAA}$-*tcdR* (MC) column purple arrowheads point to sporulating cells with mother cell-specific expression. Scale bar, 1 µm. **b**: Fractionation of spores from strains 630Δ*erm tcdR*^{FS}, *tcdR*^{MS} and Δ*tcdA*/Δ*tcdB* into a coat/exosporium and a core/cortex fractions. The proteins in the various fractions were subject to immunobloting with an anti-TcdA antibody. The position of TcdA is indicated by a black arrow.

Supplementary Figure S8

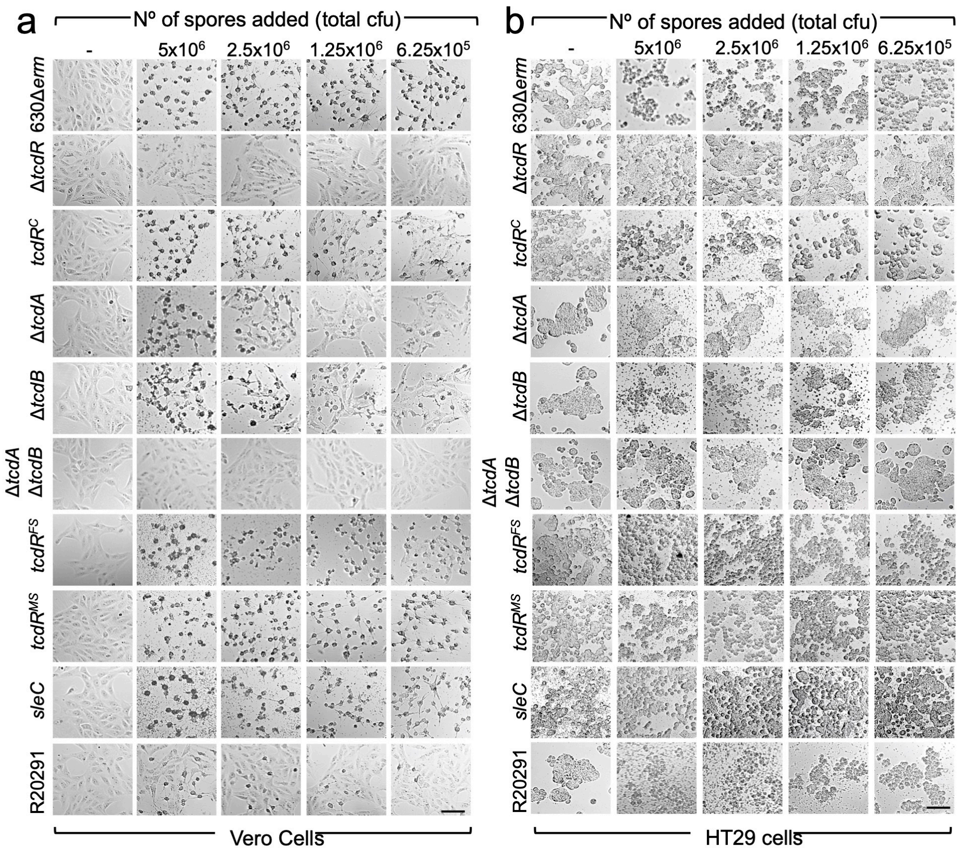

Fig. S8 - Mature spores are have a cytopathic effect. Monolayers of HT29 (**a**) and Vero (**b**) cells were exposed to different doses of purified spores (numbers at the top of the panel, in CFU´s/ml) produced by the strains indicated on the left. Following incubation, the cells were examined by phase contrast microscopy to detect cell rounding. Scale bars, 100 µm.

Supplementary Figure S9

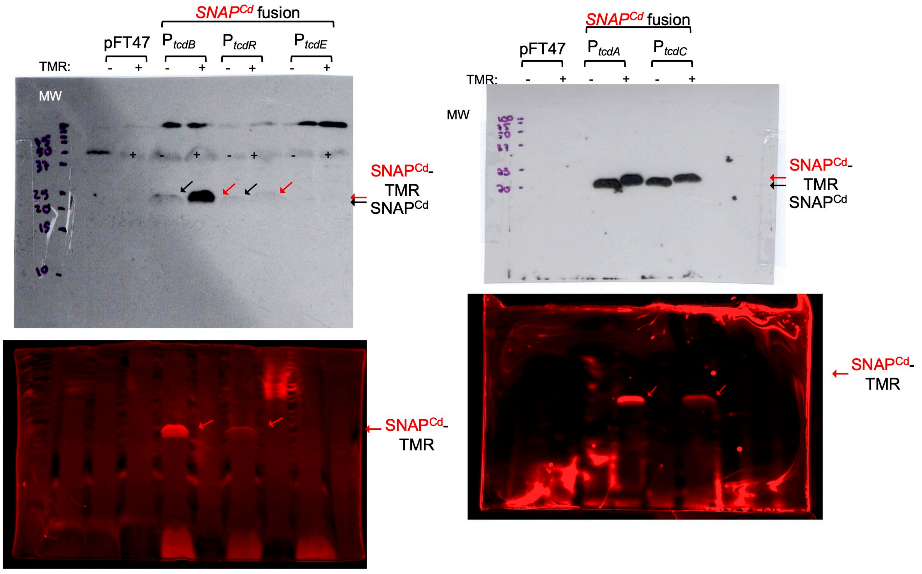

Fig. S9 - Original immunoblots and fluoroimaging scans for Figure S2. Assessing the extent of SNAP^{Cd} labeling. SDS-PAGE and western blots full-length for Figure S2. Whole cell extracts were prepared from derivatives of strain 630Δ*erm* bearing the indicated plasmids or fusions, before ("-" sign) or immediately after ("+") labeling with TMR-Star. The indicated amount of total protein was resolved by SDS-PAGE, and subject to immunoblotting with an anti-SNAP antibody (top). Duplicate gels were scanned on a fluoroimager (bottom). Black and red arrows point to unlabeled or TMR-Star-labeled, respectively, SNAP^{Cd}. Strain 630Δ*erm* carrying pFT47 (empty vector) was used as a negative control for SNAP^{Cd} production. The position of molecular weight markers (in kDa) is indicated on the left side of the panels.

Supplementary Figure S10

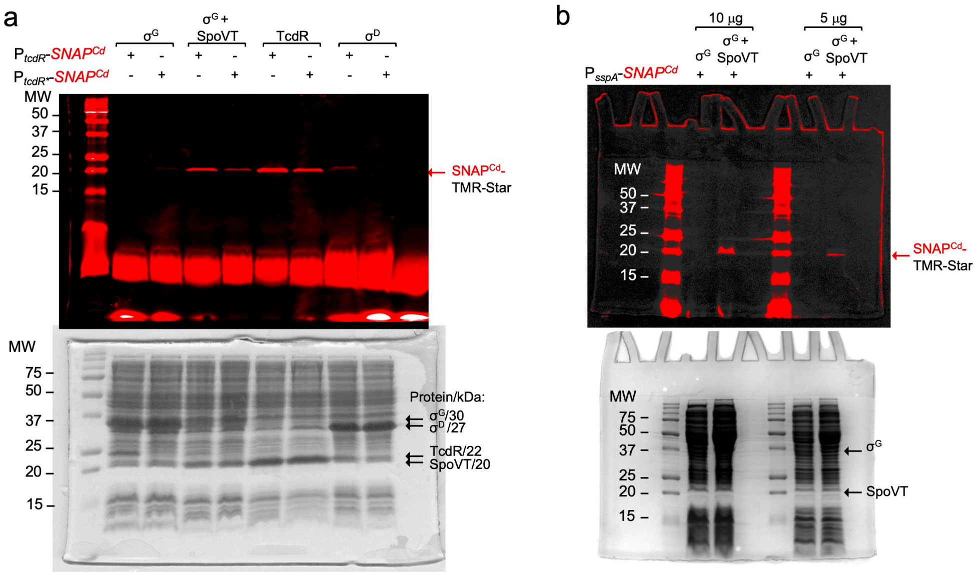

Fig. S10 - Original gels and fluoroimaging scans for Figure 4d and e. Toxin production in the forespore is dependent on σG and SpoVT. Full-length western blots for panels d and e of Figure 4. a: *E. coli* BL21(DE3) derivatives with plasmids carrying P_{tcdR}- or P_{tcdR^*}-*SNAP*^{Cd} fusions, as indicated, were transformed with compatible plasmids for the induction of σ^G, σ^G and SpoVT, TcdR or σ^D. b: As in a, except that *E. coli* BL21(DE3) containing a plasmid with a P_{sspA}-*SNAP*^{Cd} fusion was transformed with plasmids for the induction of σ^G alone or together with SpoVT, as indicated. In d and e, the various proteins were produced through auto-induction, whole cell extracts prepared, labelled with TMR-Star, proteins resolved by SDS-PAGE, and the gels scanned on a fluoroimager (top) before Coomassie staining (bottom). The position of the various regulatory proteins is indicated by black arrows and their molecular weights is given on the right side of panel a. The position of the SNAP^{Cd}-TMR-Star complex is indicated by the red arrows.

Supplementary Figure S11

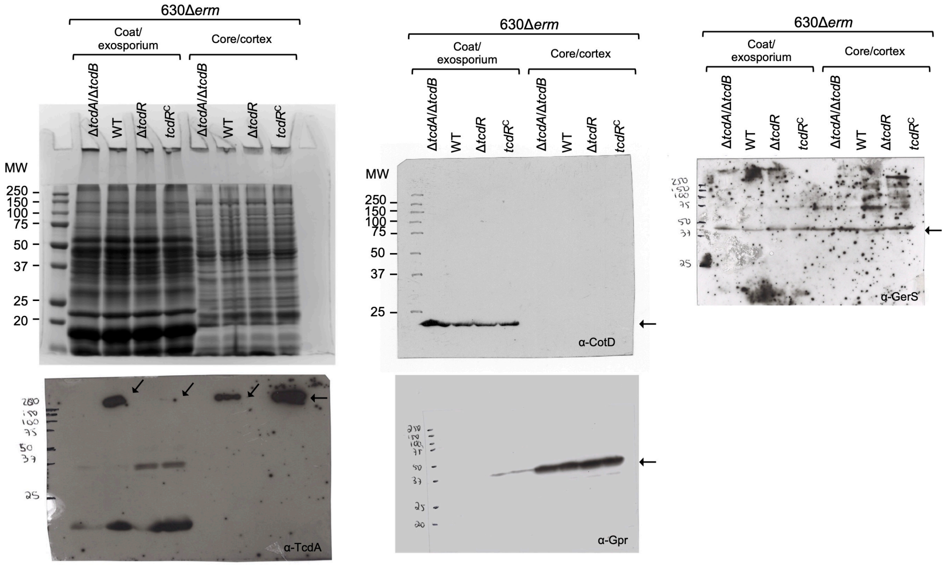

Fig. S11 - Original gels and immunoblots for Figure 5b. TcdA localization in mature spores of 630D*erm*. Full-length western blots for panel b of Figure 5. Fractionation of 630Δerm spores, their congenic ΔtcdAΔtcdB and ΔtcdR derivatives and a ΔtcdR mutant complemented with the wild-type allele at the pyrE locus, tcdRC. Intact mature spores were fractionated into a coat/ exosporium and a core/cortex fraction. The proteins in the various fractions were resolved by SDS-PAGE (top) and subject to immunoblotting (bottom) with anti-TcdA, and anti-CotD antibodies (CotD is a bona fide coat protein). Tthe extracts were also probed with anti-GPR (GPR is a germination protease localized in the spore core) and anti-GerS antibodies (GerS is a cortex-modifying protein involved in spore germination).

Supplementary Figure S12

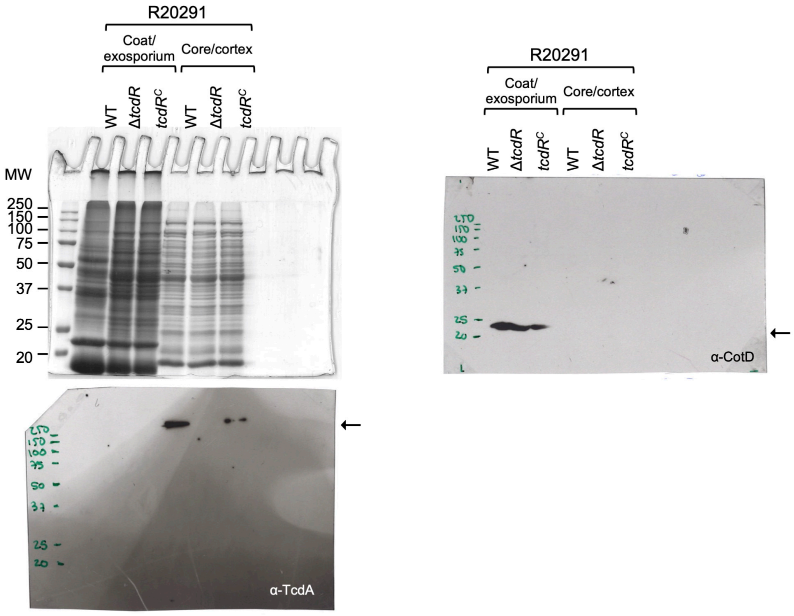

Fig. S12 - Original gels and immunoblots for Figure 5c. TcdA localization in mature spores of R20291. Full-length western blots for panel c of Figure 5. Fractionation of R20191 spores, their congenic ΔtcdAΔtcdB and ΔtcdR derivatives and a ΔtcdR mutant complemented with the wild-type allele at the pyrE locus, tcdRC. Intact mature spores were fractionated into a coat/ exosporium and a core/cortex fraction. The proteins in the various fractions were resolved by SDS-PAGE and subject to immunoblotting with anti-TcdA, and anti-CotD antibodies (CotD is a bona fide coat protein).

Supplementary Figure S13

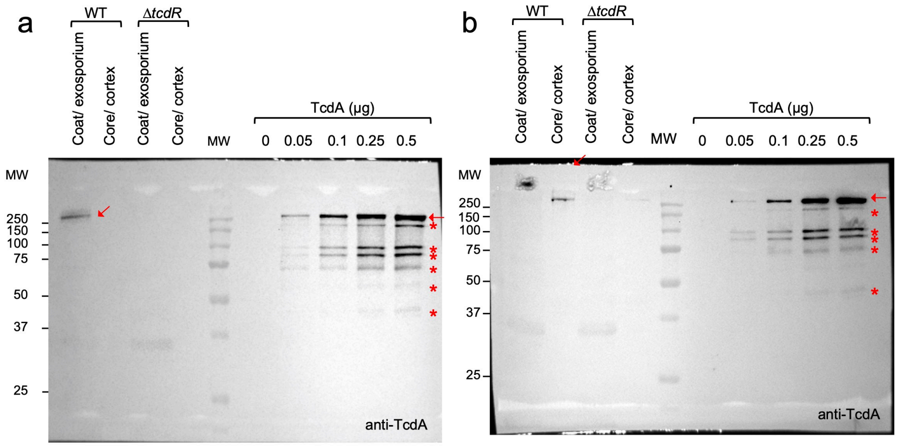

Fig. S13 - Original immunoblots for Figure S5. Quantification of spore-associated TcdA. Full-length western blots for the panels a and b of Figure S5. Quantitative immunoblotting of the spore associated TcdA toxin. Purified spores produced by strains 630Δ*erm* and Δ*tcdR* (**a**) and R20291 and a congenic Δ*tcdR* mutant (**b**) were fractionated into a coat/exosporium and a core/cortex fractions. Extracted proteins were resolved by SDS-PAGE along with increasing concentrations of purified TcdA, as indicated. The gels were then subject to immunobloting with an anti-TcdA antibody. The red arrow points to the position of full-length TcdA; other bands (red asterisks) are likely degradation products of TcdA. The position of MW markers (in kDa) is shown on the left side of the panels.

Supplementary Figure S14

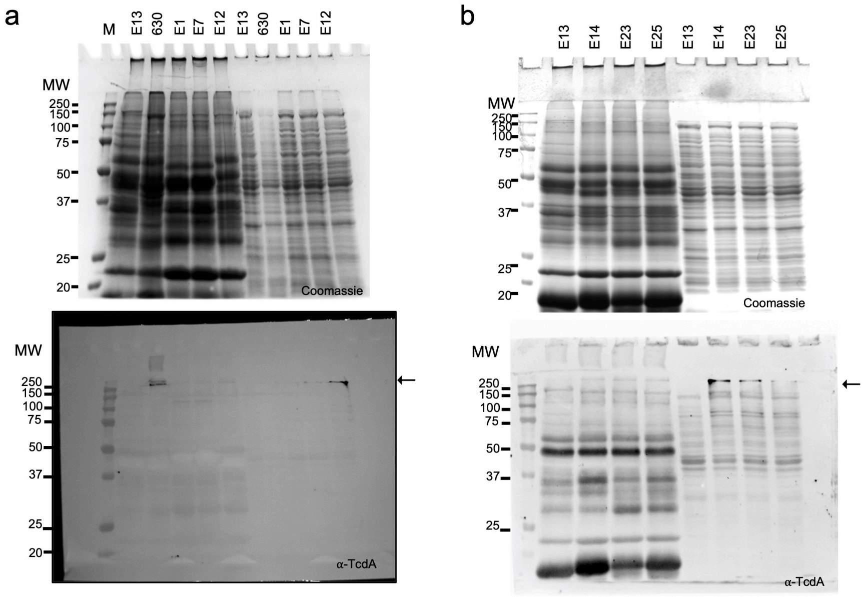

Fig. S14 - Original gels and immunoblots for Figure S6. TcdA in mature spores produced by strains of different ribotypes. Full-length SDS-PAGE stained with Coomassie and western of Figure S6. Fractionation of spores produced by the indicated strains. Purified spores were fractionated into a cortex/coat/ exosporium and a core/cortex fraction. The proteins in the various fractions were resolved by SDS-PAGE (top) and subject to immunoblotting (bottom) with anti-TcdA. Strain E13, a non-toxin producer was used as a control for the specificity of the anti-TcdA antibody. The arrows point to the position of the main form of TcdA. The position off molecular weight markers (in kDa), is shown on the left side of the panels.

Supplementary Figure S15

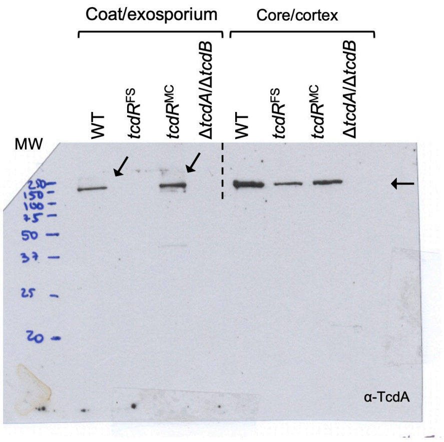

Fig. S15 - Original immunoblots for Figure S7. Forespore or mother cell-specific expression of *tcdR*. Full-length western blot for the panel b of Figure S7. Fractionation of spores from strains 630Δ*erm* $tcdR^{FS}$, $tcdR^{MS}$ and Δ*tcdA*/Δ*tcdB* into a coat/exosporium and a core/cortex fractions. The proteins in the various fractions were subject to immunobloting with an anti-TcdA antibody. The position of TcdA is indicated by a black arrow.

Supplementary Tables

Supplementary Table S1 - Heat resistant spore counts.

	630Δ*erm*			R20291		
	WT	Δ*tcdR*	Δ*tcdR*^{C}	WT	Δ*tcdR*	Δ*tcdR*^{C}
Viable	2.1±2.2x10^{9}	4.1±3.5x10^{9}	2.0±1.9x10^{9}	3.4±0.4x10^{9}	2.8±0.1x10^{9}	3.0±0.5x10^{9}
Heat	3.7±3.5x10^{4}	1.8±1.5x10^{5}	8.6±5.6x10^{4}	1.8±0.9x10^{4}	7.0±8.5x10^{4}	9.0±7.7x10^{4}

[1] the numbers represent the average and standard deviation of three different experiments (see the Material and Methods section). CFU, colony forming units.

Supplementary Table S2 - Plasmids used in this study.

Plasmid	Relevant features	Origin/ reference
pMTL84121	*Clostridium* modular plasmid containing *catP* (Cm^R /Tm^R)	Laboratory stock
pMTL-YN1	Plasmid for *pyrE* reversion through ACE in 630Δ*erm*	[2]
pMTL-YN1C	Plasmid for complementation through ACE in 630Δ*erm*	“
pMTL-YN2	Plasmid for *pyrE* reversion through ACE in R20291	“
pMTL-YN2C	Plasmid for complementation through ACE in R20291	“
pMTL-YN3	Plasmid for mutation through ACE in 630Δ*erm*	“
pMTL-YN4	Plasmid for mutation through ACE in R20291	“
pET16b	Expression vector/T7*lac* promoter	Novagen
pET28a	“	“
pETDuet-1	“	“
pFT47	pMTL84121-$SNAP^{Cd}$ (Cm^R /Tm^R)	[1]
pMS464	pFT47 containing the *tcdR* promoter region	This work
pMS470	pFT47 containing the *tcdA* promoter region	“
pMS471	pFT47 containing the *tcdB* promoter region	“
pMS472	pFT47 containing the *tcdC* promoter region	“
pMS473	pFT47 containing the *tcdE* promoter region	“
pSR1	pMTL-YN4 containing a LHA and RHA from *tcdR* (R20291)	“
pSR3	pMTL-YN3 containing a LHA and RHA from *tcdR*	“
pSR5	pMTL-YN1C containing *tcdR* and its promoter for complementation	“
pSR7	pMTL-YN3 containing a LHA and RHA from *tcdA*	“
pSR8	pMTL-YN3 containing a LHA and RHA from *tcdB*	“
pSR34	pMTL-YN2C containing *tcdR* and its promoter for complementation (R20291)	“
pFT36	pET28a containing *sigG*	“
pCC17	pET16b containing *tcdR*-Strep tagII	“
pCC20	pFT47 containing 183bp from the *tcdR* promoter region	“
pCC22	pET16b containing *spoVT*	“
pCC27	pFT47 containing the *tcdR* promoter region with point mutations at the *sigD* promoter	“
pCC29	pETDuet-1 containing *sigG* and *spoVT*	“
pCC30	pET28a containing *sigD*	“
pCC32	pFT47 containing the *sspA* promoter region	“
pCC44	pMTL-YN1C containing *tcdR* with optimized RBS	“
pCC45	pCC44 containing the *sspA* promoter	“
pCC46	pCC44 containing the *spoIIIAA* promoter	“
pCC86	pMTL-Cas9 containing a LHA and RHA from *spoVT* and the sgRNA with the SEED sequence for *spoVT*	“

Supplementary Table S3 - Bacterial strains used in this study.

Strain	Relevant properties	Origin/ reference
E. coli		
DH5α	General cloning host	Invitrogen
HB101 (RP4)	Host for conjugation into *C. difficile*	Laboratory stock
AHEC068	BL21 (DE3)/ pFT36	This work
AHEC076	HB101 (RP4)/ pMTL84121	[9]
AHEC125	HB101 (RP4)/ pMS464	This work
AHEC144	HB101 (RP4)/ pMS470	“
AHEC145	HB101 (RP4)/ pMS471	“
AHEC146	HB101 (RP4)/ pMS472	“
AHEC147	HB101 (RP4)/ pMS473	“
AHEC203	HB101 (RP4)/ pMTL-YN1	[2]
AHEC205	HB101 (RP4)/ pMTL-YN2	“
AHEC217	Bl21 (DE3)/ pCC17	This work
AHEC227	HB101 (RP4)/ pSR1	“
AHEC237	HB101 (RP4)/ pCC20	“
AHEC239	HB101 (RP4)/ pSR3	“
AHEC274	HB101 (RP4)/ pCC27	“
AHEC285	HB101 (RP4)/ pSR5	“
AHEC290	BL21 (DE3)/ pFT36/ pMS464	“
AHEC291	BL21 (DE3)/ pFT36/ pCC27	“
AHEC304	BL21 (DE3)/ pCC29	“
AHEC313	BL21 (DE3)/ pCC30	“
AHEC314	BL21 (DE3)/ pCC29/ pMS464	“
AHEC316	BL21 (DE3)/ pCC29/ pCC27	“
AHEC317	BL21 (DE3)/ pCC17/ pMS464	“
AHEC319	BL21 (DE3)/ pCC17/ pCC27	“
AHEC320	BL21 (DE3)/ pCC30/ pMS464	“
AHEC389	HB101 (RP4)/ pCC46	“
AHEC391	HB101 (RP4)/ pCC45	“
AHEC498	HB101 (RP4)/ pSR7	“
AHEC499	HB101 (RP4)/ pSR8	“
AHEC661	HB101 (RP4)/ pSR34	“
AHEC853	BL21 (DE3)/ pFT36/ pCC32	“
AHEC855	HB101 (RP4)/ pCC86	“
AHEC859	BL21 (DE3)/ pCC29/ pCC32	“
C. difficile		
AHCD531	*C. difficile* 630Δerm	[10]
AHCD532	*630Δerm sigE*::intron *ermB**	[1]
AHCD533	*630Δerm sigF*::intron *ermB*	“
AHCD534	630Δ*erm sigG*::intron *ermB*	“
AHCD535	630Δ*erm sigK*::intron *ermB*	“
AHCD536	630Δ*erm spo0A*::intron *ermB*	[11]
AHCD543	630Δ*erm* containing pMTL84121	[1]
AHCD608	630Δ*erm* containing pMS464	This work
AHCD647	AHCD536 containing pMS464	“
AHCD648	AHCD533 containing pMS464	“
AHCD649	AHCD532 containing pMS464	“
AHCD650	AHCD534 containing pMS464	“
AHCD651	AHCD535 containing pMS464	“
AHCD668	630Δ*erm* containing pMS470	“
AHCD669	630Δ*erm* containing pMS471	“
AHCD670	630Δ*erm* containing pMS472	“
AHCD671	630Δ*erm* containing pMS473	“
AHCD687	630Δ*erm sigD*::intron *ermB*	[12]
AHCD688	*C. difficile* R20291	Anaerobe Reference Laboratory, Cardiff, Wales, United Kingdom
AHCD772	630Δ*erm* Δ*pyrE*	[2]
AHCD777	AHCD659 containing pMS464	This work
AHCD779	AHCD687 containing pMS470	“

AHCD780	AHCD687 containing pMS464	“
AHCD795	630Δ*erm* containing pCC20	“
AHCD811	630Δ*erm* Δ*pyrE* Δ*tcdR*	“
AHCD820	630Δ*erm* Δ*tcdR*	“
AHCD821	630Δ*erm* containing pCC27	“
AHCD828	630Δ*erm* Δ*tcdR pyrE::tcdR*	“
AHCD840	AHCD820 containing pMS470	“
AHCD841	AHCD820 containing pMS464	“
AHCD845	AHCD828 containing pMS470	“
AHCD846	AHCD828 containing pMS464	“
AHCD900	630Δ*erm* Δ*tcdR pyrE::PsspA-tcdR*	“
AHCD926	630Δ*erm* Δ*tcdR pyrE:: PspoIIIAA-tcdR*	“
AHCD999	630Δ*erm* Δ*pyrE* Δ*tcdA*	“
AHCD1002	630Δ*erm* Δ*tcdA*	“
AHCD1046	630Δ*erm* Δ*tcdA* Δ*tcdB* Δ*pyrE*	“
AHCD1066	630Δ*erm* Δ*tcdA* Δ*tcdB*	“
AHCD1067	R20291 Δ*pyrE*	[2]
AHCD1093	R20291 Δ*pyrE* Δ*tcdR*	This work
AHCD1102	R20291 Δ*tcdR*	“
AHCD1161	R20291 Δ*tcdR pyrE::tcdR*	“
AHCD1415	630Δ*erm* Δ*pyrE* Δ*tcdB*	“
AHCD1418	630Δ*erm sigG spoVT*	“
AHCD1439	630Δ*erm* Δ*tcdB*	“
E1	Ribotype 126	[13]
E7	Ribotye 053	“
E12	Ribotype 106	“
E13	Ribotype 017	“
E14	Ribotype 014	“
E23	Ribotype 001/072	“
E25	Ribotype 005	“

**ermB*, erythromycin resistance determinant.

Supplementary references

1. Pereira, F. C. *et al.* The spore differentiation pathway in the enteric pathogen Clostridium difficile. *PLoS genetics* **9**, e1003782–e1003782 (2013).
2. Ng, Y. K. *et al.* Expanding the repertoire of gene tools for precise manipulation of the Clostridium difficile genome: allelic exchange using pyrE alleles. *PloS one* **8**, e56051–e56051 (2013).
3. Karasawa, T., Ikoma, S., Yamakawa, K. & Nakamura, S. A defined growth medium for Clostridium difficile. *Microbiology* **141**, 371–375 (1995).
4. Ingle, P. *et al.* Generation of a fully erythromycin-sensitive strain of Clostridioides difficile using a novel CRISPR-Cas9 genome editing system. *Scientific Reports* **9**, 8123–8123 (2019).
5. Permpoonpattana, P. *et al.* Functional characterization of Clostridium difficile spore coat proteins. *Journal of Bacteriology* **195**, 1492–1503 (2013).
6. Fimlaid, K. A. *et al.* Global analysis of the sporulation pathway of Clostridium difficile. *PLoS genetics* **9**, e1003660–e1003660 (2013).
7. Diaz, O. R., Sayer, C. V., Popham, D. L. & Shen, A. Clostridium difficile Lipoprotein GerS Is Required for Cortex Modification and Thus Spore Germination. *mSphere* **3**, e00205-18 (2018).
8. Yang, G. *et al.* Expression of recombinant Clostridium difficile toxin A and B in Bacillus megaterium. *BMC Microbiol* **8**, 192 (2008).
9. Heap, J. T., Pennington, O. J., Cartman, S. T. & Minton, N. P. A modular system for Clostridium shuttle plasmids. *Journal of Microbiological Methods* **78**, 79–85 (2009).
10. Hussain, H. A., Roberts, A. P. & Mullany, P. Generation of an erythromycin-sensitive derivative of Clostridium difficile strain 630 (630Δerm) and demonstration that the conjugative transposon Tn916ΔE enters the genome of this strain at multiple sites. *Journal of Medical Microbiology* **54**, 137–141 (2005).
11. Saujet, L., Monot, M., Dupuy, B., Soutourina, O. & Martin-Verstraete, I. The Key Sigma Factor of Transition Phase, SigH, Controls Sporulation, Metabolism, and Virulence Factor Expression in Clostridium difficile. *J Bacteriol* **193**, 3186–3196 (2011).

12. El Meouche, I. *et al.* Characterization of the SigD Regulon of Clostridium difficile and Its Positive Control of Toxin Production through the Regulation of tcdR. *PLoS One* **8**, (2013).
13. Kurka, H. *et al.* Sequence Similarity of Clostridium difficile Strains by Analysis of Conserved Genes and Genome Content Is Reflected by Their Ribotype Affiliation. *PLOS ONE* **9**, e86535 (2014).
