# Supplementary material for: Spores of Clostridioides difficile are toxin delivery vehicles
Source: Commun Biol. 2024 Jul 10;7:839. doi: 10.1038/s42003-024-06521-x (PMC11237016; doi:10.1038/s42003-024-06521-x)
Supplement: Supplementary file 2 — Description of additional supplementary files [file 42003_2024_6521_MOESM2_ESM.docx]

Description of Additional Supplementary Files

**File name:** Supplementary Data

**Description:** Source data for Figures 2b, 3c, 4c and 6b
